# The economic burden of antibiotic resistance: A systematic review and meta-analysis

**DOI:** 10.1371/journal.pone.0285170

**Published:** 2023-05-08

**Authors:** Ak Narayan Poudel, Shihua Zhu, Nicola Cooper, Paul Little, Carolyn Tarrant, Matthew Hickman, Guiqing Yao

**Affiliations:** 1 School of Human and Health Sciences, University of Huddersfield, Huddersfield, England, United Kingdom; 2 Primary Care, Population Sciences and Medical Education, University of Southampton, Southampton, England, United Kingdom; 3 Department of Health Sciences, University of Leicester, Leicester, England, United Kingdom; 4 Bristol Medical School, Population Health Sciences, University of Bristol, Bristol, England, United Kingdom; Nitte University, INDIA

## Abstract

**Introduction:**

Antibiotic resistance (ABR) has substantial global public health concerns. This systematic review aimed to synthesise recent evidence estimating the economic burden of ABR, characterised by study perspectives, healthcare settings, study design, and income of the countries.

**Methods:**

This systematic review included peer-reviewed articles from PubMed, Medline, and Scopus databases, and grey literature on the topic of the economic burden of ABR, published between January 2016 and December 2021. The study was reported in line with ‘Preferred Reporting Items for Systematic Review and Meta-Analysis’ (PRISMA). Two reviewers independently screened papers for inclusion first by title, then abstract, and then the full text. Study quality was assessed using appropriate quality assessment tools. Narrative synthesis and meta-analyses of the included studies were conducted.

**Results:**

A total of 29 studies were included in this review. Out of these studies, 69% (20/29) were conducted in high-income economies and the remainder were conducted in upper-and-middle income economies. Most of the studies were conducted from a healthcare or hospital perspective (89.6%, 26/29) and 44.8% (13/29) studies were conducted in tertiary care settings. The available evidence indicates that the attributable cost of resistant infection ranges from -US$2,371.4 to +US$29,289.1 (adjusted for 2020 price) per patient episode; the mean excess length of stay (LoS) is 7.4 days (95% CI: 3.4–11.4), the odds ratios of mortality for resistant infection is 1.844 (95% CI: 1.187–2.865) and readmission is 1.492 (95% CI: 1.231–1.807).

**Conclusion:**

Recent publications show that the burden of ABR is substantial. There is still a lack of studies on the economic burden of ABR from low-income economies, and lower-middle-income economies, from a societal perspective, and in relation to primary care. The findings of this review may be of value to researchers, policymakers, clinicians, and those who are working in the field of ABR and health promotion.

**Systematic review registration:**

CRD42020193886

## Introduction

Global health has improved significantly since the discovery of penicillin in 1928. However, organisms, such as bacteria have become increasingly resistant to many antibiotics in recent years [1]. Although antibiotic resistance (ABR) is a natural phenomenon, the process of resistance is accelerated by overuse and misuse of antibiotics, and intercontinental travel [2–4]. In the past, resistant infections were associated with hospitals and care settings, but over the last decades, these have been spread in the wider community [5]. In addition, human consumption of antibiotics across the world has increased by nearly 40% in a decade (between 2000 and 2010) [2]. There is considerable variation in ABR problems in individual countries, which depends on how heavily they use antibiotics [2]. Evidence shows that low-and middle-income countries (LMICs) are still far behind high-income countries (HICs) in terms of suppressing the spread of ABR [6, 7].

Antibiotic resistance (ABR) is one of the most challenging health problems faced by every country across the world [3]. It has been estimated that the burden of deaths by antibiotic resistance may increase to 10 million each year by 2050, if action is not taken now [5]. In Europe and the United States of America (USA) alone, antimicrobial resistance (AMR) has claimed over 50,000 lives each year, with thousands more dying in other countries around the globe [2]. If effective actions are not taken, ABR may claim around 2.4 million lives in Europe, North America and Australia between 2015 and 2050 [8]. AMR might impact 1.1% of gross domestic product (GDP) reduction and may exceed US$ one trillion annually after 2030 across the globe, in a low-impact scenario [9]. In G20 countries such as the Russian Federation, China and India, over 40% of infections were caused by resistant bacteria and about 17% in OECD countries [8]. Moreover, antibiotics use in agriculture, especially in livestock, is significant across the world. In 2010 alone, an estimated 63,200 tons of antibiotics were used in livestock across the globe, possibly far more than total human consumption [9]. In the USA alone, over 70% of antibiotics (by weight) are sold for livestock use compared to for humans [5]. However, many countries do not hold information on antibiotics use in farming, and detailed impacts of antibiotics use in agriculture, environment, human health, and economy are scarce.

From a health economics perspective, patients with ABR infections use more resources for their treatment as they generally have worse clinical outcomes compared to patients with non-resistant infection [3]. Majority of the past studies show that the healthcare cost for patients with resistant infections is higher than the care for patients with non-resistant infections because of longer duration of illness, additional diagnostic tests, longer hospital stays, need for more expensive drugs, and increased mortality [3, 4]. Therefore, accurate estimation of the burden of ABR is important to prevent the spread of the ABR bacteria and allocate resources reasonably to control the ABR. Moreover, the availability of reliable evidence and a better understanding of the health and economic impact of ABR can support the formulation of priority interventions for public health promotion.

Although several rapid reviews on the economic burden of ABR have been conducted in the past [2, 10–24]; adequate reporting and transparency are always a concern [25]. For example, most of the rapid reviews neither assessed the quality of the included papers nor followed standard reporting procedures (such as Preferred Reporting Items for Systematic Review and Meta-Analysis (PRISMA) checklist). In the last 20 years, only five systematic reviews have been published on the economic burden of ABR or AMR looking at different aspects [6, 26–29]. These previous systematic reviews did not differentiate the economic burden of antibiotic resistance by the income of the countries, healthcare settings or study designs.

This systematic review aimed to synthesise, summarise and critique the recent relevant literature in estimating the economic burden of ABR to address the following research questions:

- What perspectives and methodologies have been used to estimate the economic consequences of ABR in the recently published literature?
- How do the economic consequences of ABR differ by study perspectives (patient, healthcare-wide, or societal), healthcare settings and income of the countries?
- To what extent does the published literature in recent years provide high-quality evidence on the economic burden of ABR?

## Methods/design

This review is in line with the ‘Preferred Reporting Items for Systematic Review and Meta-Analysis’ (PRISMA) checklist [30] and is registered to the International Prospective Register of Systematic Reviews (PROSPERO) with the registration number- CRD42020193886. Ethical approval was not required for this study.

### Search strategy and eligibility criteria

Studies related to economic burden of ABR around the world, published from January 2016 to December 2021, were included in this review. Searches were carried out in PubMed, Medline, Scopus databases and grey literature. Search strategies were developed based on search terms identified from previous relevant studies [28, 31] and were adapted to match the requirements of different databases. Terms included were: excess, attributable, associated, burden, morbidity, mortality, cost, economic, clinical, global, resistan*, multidrug, susceptib*, nonsusceptib*, enterococc*, Escherichia, streptococc*, staphylococc*, klebsiella, pseudomonas, Neisseria, chlamydia, clostridia*, mycobacteri*, gram-positive, and gram-negative. The reference lists of eligible studies were also reviewed to identify additional papers. In addition to the above, the following websites were searched to identify grey literature: UK Health Security Agency (UKHSA), Office for Health Improvement and Disparities (OHID), Public Health Wales (PHW), Health Protection Scotland (HPS), Public Health Scotland (PHS), Department of Health and Social Care (DHSC) (UK), Health Protection Agency (HPA), National Institute for Health and Care Excellence (NICE), Centre for Disease Control and Prevention (CDC), World Health Organisation (WHO), Public Health Europe (PHE) and Review on Antimicrobial Resistance (AMR Review).

In this review, the studies were selected by using the pre-specified eligibility criteria as shown in the Table 1.

**Table 1:** Eligibility criteria and PICO.

Inclusion criteria	Exclusion criteria	PICO
Studies on economic consequences or burden of ABR (e.g., healthcare cost, societal cost, treatment costs) or studies related to economic evaluations or modelling having relevant information (such as per patient costs for treatment due to ABR) Studies comparing ‘resistant’ with ‘susceptible’ cases or study on ‘resistant cases’ with other suitable comparator Original case control, cohort, observational, cross-sectional, longitudinal, RCT, or modelling studies Studies on human related to ABR Studies published in English language Studies published from January 2016 to December 2021.	Studies not related to economic consequences or burden of ABR (e.g., not having information on healthcare cost, societal cost, treatment costs) or studies related to economic evaluations or modelling but not having relevant information, e.g., not reporting treatment cost of ABR per patient Theoretical studies which do not calculate the burden of ABR Studies which report only health outcomes (e.g., mortality, morbidity) Studies related to epidemiology only or molecular biology only Studies not comparing ‘resistant’ cases with ‘susceptible’ cases or not having suitable comparator Reviews, letters, notes, editorials and conference reports Study related to ABR on animals or plants Not published in English language Studies published before January 2016	**1. Population:** Human All sexes All ages Infected with resistance or susceptible infections **2. Intervention:** Not applicable **3. Comparison:** Antibiotic resistance infections versus antibiotic susceptible infections or other suitable comparator **4. Outcome:** **Economic burdens** such as health care cost, opportunity costs, length of stay, mortality, readmission by study perspective, healthcare settings, study type and income of countries

### Data extraction, quality assessment and reporting

**Fig 1:**
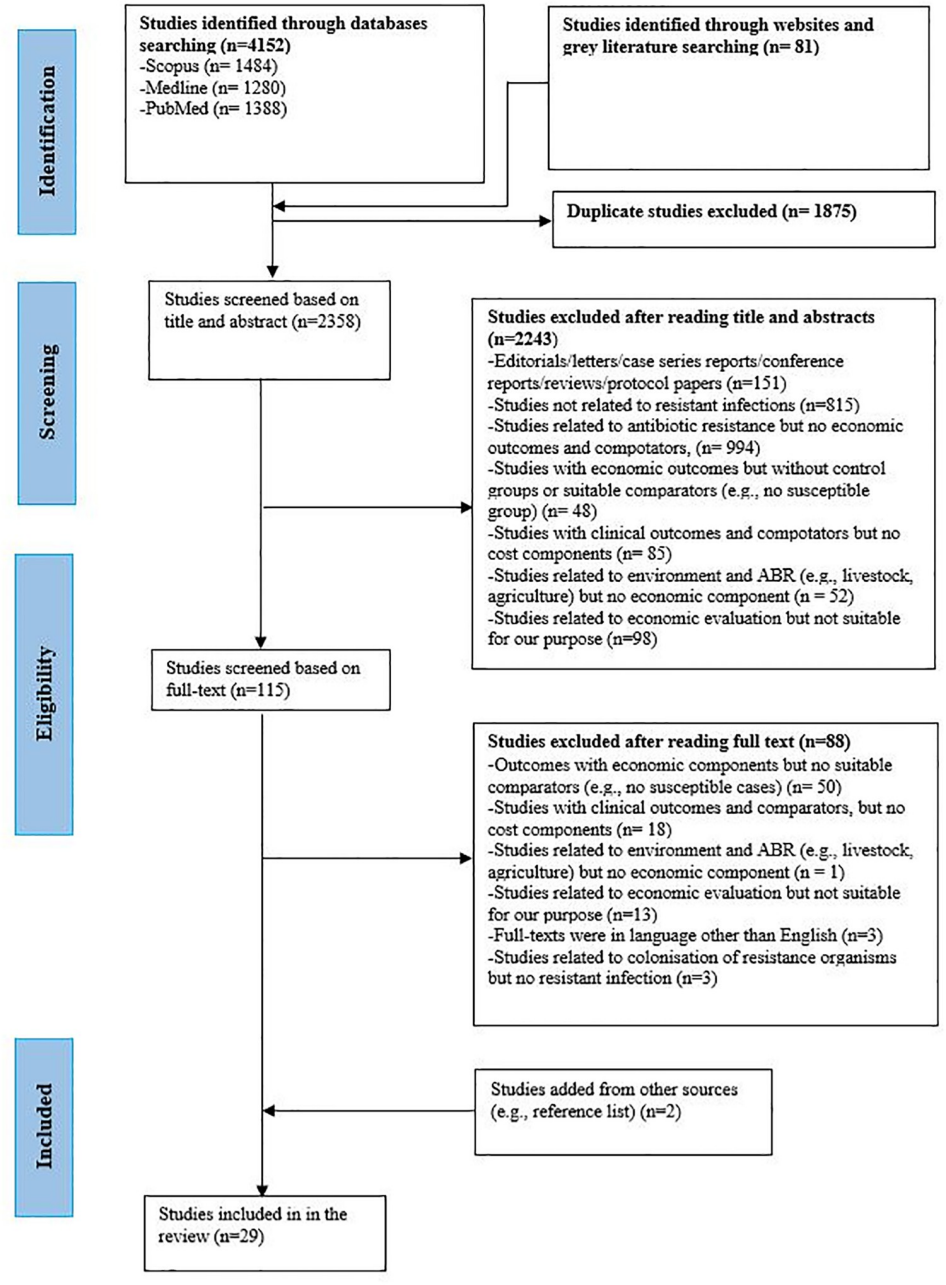
PRISMA flow diagram.

The PRISMA flow diagram showing the study selection processes is provided in Fig 1. Data were extracted by the lead reviewer (ANP) and inputted into a standardised data extraction table in Microsoft Excel. The table was developed and piloted with five eligible papers, and necessary revisions were made. The quality of extracted data was independently checked by two reviewers (ANP and SZ). The following data were extracted from the selected studies: general information about the studies such as first author and publication year, study period, study title, study objectives, study country, income category of the country, study centre (single centre, multi-centre or national or multi-national), study settings (primary, secondary or tertiary hospitals), type of study/study design, study perspectives (patients/payers, health care/ healthcare system, societal), study population, sample size, and data related to economic outcome indicators (e.g. cost of treatment, length of stay). Regarding costs-related data, we extracted the currency with cost year, mean or median total costs (for both case and control groups) and attributable costs due to resistance infections if reported. Likewise, data related to clinical outcome indicators (e.g., mortality, readmissions), data related to study methodologies and statistical analysis of cost data (such as regression analysis, survival analysis, matching, multistate models, economic model, stepwise, sensitivity analysis, significance tests) and stated limitations and study quality were also extracted. As methodologies differ in modelling and other studies (such as descriptive or significance test), we extracted methodology related information in detail. We recorded the information related to the matching of case group with a control group or not, whether they had considered confounding factors or not in their analysis, what methods were used to reduce confounding effects (e.g., propensity score matching (PSM), Carlson comorbidity index), and time dependent biases (e.g., multistate model), and what types of analyses were conducted in each type of study. Moreover, we also extracted data about methods of modelling used (e.g., multistate model, state transition model, decision tree model). Data on outcomes (i.e., results) may have differed greatly according to the methods used in the studies. We carefully considered variations in the methodologies in different studies and extracted the data accordingly.

The risk of bias or quality of the studies were assessed by two reviewers (ANP and SZ) using appropriate tools. The quality of the non-randomised studies, such as cohort and case-control, were assessed using the Newcastle-Ottawa Quality Assessment Scale (NOS). NOS uses three dimensional criteria to assess the quality of the methodology: a) selected population, b) comparability of groups, and c) outcome or exposure of interests [32]. We used the Philips checklist to assess the quality of the modelling and economic evaluation studies [33]. There were no randomised control trials (RCTs) in our reviewed papers. However, we had three cross-sectional studies; for these we used the Appraisal tool for Cross-Sectional Studies (AXIS tool) [34]. For the comparison between studies, we converted the stars into a score between zero (0) (achieved no star at all) to one (achieved all possible stars) by dividing achieved stars by possible stars, as reported in Naylor et al. (2018) [28]. The risk of bias across the evidence was presented using median and interquartile ranges of the quality score by the method of analysis. The quality assessments of the included studies were done independently by ANP and SZ. The risk of bias was assessed within the study and across the studies (e.g., Funnel Plot, Duval and Tweedie’s trim and fill). Where required, clinical experts (PL and MH) were consulted; for example, to answer queries regarding the types of resistant and susceptible infections, or categories of health care settings. Endnote was used to manage the records and references.

### Data analysis

We used Comprehensive Meta-Analysis (CMA) software along with Microsoft Excel to analyse extracted data. We performed Meta-Analyses to assess the effect of the independent variable (status of antibiotic resistant infection: resistant and susceptible infections) on outcome variables (such as length of stay, mortality and readmission). In addition, we also conducted sub-group analysis using moderator or grouping variables including healthcare settings (primary, secondary and tertiary), study perspectives (e.g., patient, healthcare or hospital or societal), types of studies (e.g., cohort, case-control, cross-sectional) and income category of countries [(according to the World Bank classification: low-income economies (LIE), lower-middle income economies (LMIE), upper-middle income economies (UMIE), high-income economies (HIE)]) [35]. We did not perform Meta-Analysis for healthcare cost because of high level of variations among the studies in estimating the cost (e.g., cost categories included), therefore we conducted a narrative synthesis in accordance with the Cochrane Handbook guidance [36]. If there was more than one healthcare cost, length of stay, mortality, or readmission data for resistant and susceptible infections included in an individual study, we chose the most relevant to our review. For example, we chose adjusted data over unadjusted, we chose overall mean estimates over individual means and so on. For those studies that did not report mean and standard deviation (SD) but provided median and interquartile range (IQR) or range (e.g. for our outcome variables costs, length of stay (LoS)) these data were converted into mean and standard deviation using methods suggested in the published literature [37–40].

All the costs data for the studies conducted before 2020 and in local currencies, were adjusted using GDP deflator data by the World Bank [41], and where necessary converted in to US dollars using the World Bank exchange rate for 2020 [42]. These adjustments of the cost data were in line with the methodology suggested by Turner et al. (2019) [43]. Total cost in this study is defined as the sum of the bed charges, cost for medicines, cost for staff and diagnostic tests. However, when the costs were calculated from societal perspective, it would include productivity loss of patients and costs for carer in addition to above. Likewise, attributable cost is defined as the difference of healthcare cost between resistant and susceptible infection (i.e., healthcare cost of resistant infection minus healthcare cost of susceptible infection). The attributable cost can be negative when healthcare cost of susceptible infection is higher than resistant infection.

We used the random effects Meta-Analysis models to allow for heterogeneity in the treatment effect between studies [44] which may be caused by differences in study populations, study design, healthcare settings, perspectives, countries and other factors [45]. We also conducted fixed-effects Meta-Analysis models where there was no heterogeneity (e.g., for hospital readmission). We have presented forest plots with individual effect size (Hedges’s g, odds ratios) of each study, combined effect size and sub-group effect sizes (where possible) together with 95% confidence interval. Results are reported below according to the interpretation guidelines for PRISMA [30] and Meta-Analysis [46].

## Results

A total of 4233 studies from Scopus (1484 results), Medline (1280 results), PubMed (1388 results) and grey literature (81 results) were retrieved. After applying eligibility criteria, a total of 29 studies were included in this systematic review (Fig 1). As reported in Table 2, 20 out of the 29 studies (69%) were conducted in high income economies (HIE) [47–65] and rest (n = 9, 31%) were conducted in upper-middle income economies (UMIE) [66–74], but there were no studies from low-income economies (LIE). The countries which produced the highest number of studies were the USA (n = 10, 34.5%) [50, 51, 53–55, 58, 60, 65, 75, 77], followed by China (n = 7, 24.1%) [66, 67, 69–73] and Japan (n = 3, 10.3%) [47–49].

**Table 2:** Characteristics of the included studies: Sample size, studied bacteria, site of infection, outcomes of interest, and analysis methods.

Ref no.	First author & publication year	Study country, Single or multicentre, sample size for resistant and susceptible infections	Exposure group	Control group	Site of infection, study design, healthcare settings & perspective of the study	Outcome of interest, method of analysis
[70]	Huang et al. 2018	China, Single centre, Resistant: 267 Susceptible: 1328	Carbapenem resistance in *K*. *pneumoniae*	Carbapenem susceptible in *K*. *pneumonia*	Multiple sites Case- control study (retrospective) Tertiary hospital Healthcare/ hospital	Healthcare costs, length of stay, mortality rate, Regression
[67]	Jiang et al. 2017	China, Taiwan, Single centre, Resistant: 48 Susceptible: 142	Vancomycin-resistant Enterococcus (VRE) infections	Vancomycin-susceptible Enterococcus (VSE) infections	Multiple sites (Skin, would, urinary tract, intestines)- mainly in fluids Case- control study Not reported Healthcare/ hospital	Healthcare costs, length of stay, mortality (odds ratio), Survival analysis
[76]	Klein et al. 2019	USA, National level, Resistant: 358140 Susceptible: 257930	MRSA	MSSA	Multiple sites Cohort study (retrospective) Secondary + tertiary hospitals Healthcare/ hospital	Healthcare costs, length of stay, mortality rate Matching
[62]	Maslikowska et al. 2016	Canada, Single centre, Resistant: 75 Susceptible: 75	Extended-spectrum b-lactamase (ESBL)-producing Escherichia coli and Klebsiella spp.	ESBL-negative E. coli and Klebsiella spp.	Multiple sites Case- control study (retrospective) Tertiary hospital Healthcare/ hospital	Healthcare costs, length of stay, mortality rate Matching
[72]	Meng et al. 2017	China, Single centre, Resistant: 49 Susceptible: 98	Carbapenem-resistant Escherichia coli (CREC)	Carbapenem- susceptible Escherichia coli (CREC)	Multiple sites Case- control study (retrospective) Tertiary hospital Healthcare/ hospital	Healthcare costs, mortality rate Regression
[57]	Naylor et al. 2019	UK (England), National level Resistant: 14042 Susceptible: 8919275	Resistant E. coli	Susceptible E. coli	Blood stream Cohort study (retrospective) Secondary healthcare setting Healthcare/ hospital	Attributable costs, length of stay, Multistate models
[63]	Puchter et al. 2018	Germany, Single centre, Resistant: 42 Susceptible: 42	Vancomycin-resistant enterococci (VRE) infections (E. faecium and E. faecalis) (HAI infection)	Vancomycin-susceptible enterococci (VSE) infections (E. faecium and E. faecalis) (HAI infection)	Multiple sites Case- control study (retrospective) Tertiary hospital Healthcare/ hospital	Healthcare costs, length of stay, mortality rate Regression
[60]	Thaden et al. 2017	USA, Single centre, Resistant: 292 Susceptible: 599	All MDR bloodstream infections (BSI) gram-negative bacteria	Non-MDR BSI gram-negative bacteria	Blood stream Cohort study (prospective) Tertiary hospital Healthcare/ hospital	Healthcare costs, length of stay, mortality (odds ratio) Regression
[58]	Judd et al. 2016	USA, Single centre, Resistant: 32 Susceptible: 350	Resistant P. aeruginosa	Susceptible P. aeruginosa	Multiple sites Cohort study (retrospective) Tertiary hospital Healthcare/ hospital	Healthcare costs, length of stay, mortality (odds ratio) Regression
[64]	Stewardson et al. 2016	10 European hospital: Italy (3), Germany (2), France (1), Spain (1), UK (2) and Switzerland (1), Resistant: 523 Susceptible: 2985	Resistance Escherichia coli, Klebsiella spp. or Proteus spp. Strains, methicillin resistance among Staphylococcus aureus and resistance to third generation cephalosporins among Enterobacteriaceae	Susceptible Escherichia coli, Klebsiella spp. or Proteus spp. Strains, methicillin susceptible Staphylococcus aureus and susceptible to third generation cephalosporins among Enterobacteriaceae	Blood stream Cohort study (retrospective) Secondary + tertiary hospitals Healthcare/ hospital	Healthcare costs, mortality rate Multistate models
[51]	Tabak et al. 2019	USA, Multi-centre, Resistant: 1429 Susceptible: 7145	Gram-negative isolates that tested as ‘resistant’ or ‘intermediate’ to imipenem or meropenem for P. aeruginosa and A. baumannii, Enterobacteriaceae (E. coli, Klebsiella pneumoniae, P. mirabilis, E. cloacae, Enterobacter aerogenes, Serratia marcescens, Citrobacter freundii, Morganella morganii) were classified as C-NS	All the organisms reported in exposure groups and tested as ‘susceptible’ to imipenem or meropenem for P. aeruginosa and A. baumannii, Enterobacteriaceae (E. coli, Klebsiella pneumoniae, P. mirabilis, E. cloacae, Enterobacter aerogenes, Serratia marcescens, Citrobacter freundii, Morganella morganii) were classified as C-S	Urinary tract Cohort study (retrospective) Acute care hospitals Healthcare/ hospital	Healthcare costs, length of stay, mortality (odds ratio), readmission (odds ratio) Regression
[75]	Tabak et al. 2020	USA, Multi-centre Resistant: 1348 Susceptible: 1348	Gram-negative carbapenem-non-susceptible pathogens, e.g.: Pseudomonas aeruginosa or Acinetobacter baumannii, Escherichia coli, Klebsiella pneumoniae, Proteus mirabilis, Enterobacter cloacae, Enterobacter aerogenes, Serratia marcescens, Citrobacter freundii, Morganella morganii	Gram negative carbapenem-susceptible pathogens: Pseudomonas aeruginosa or Acinetobacter baumannii, Escherichia coli, Klebsiella pneumoniae, Proteus mirabilis, Enterobacter cloacae, Enterobacter aerogenes, Serratia marcescens, Citrobacter freundii, Morganella morganii	Respiratory infections Cohort study (retrospective) Acute care hospitals Healthcare/ hospital	Healthcare costs, length of stay, mortality (odds ratio), readmission (odds ratio) Regression
[68]	Thatrimontrichai et al. 2019	Thailand, Single centre, Resistant: 157 Susceptible: 218	Multidrug-resistant Gram-negative bacilli: *Enterobacteriaceae (gram-negative)* and some non-*Enterobacteriaceae* species (*Pseudomonas aeruginosa* and *Acinetobacter* spp.)	Non- Multidrug-resistant Gram-negative bacilli: *Enterobacteriaceae (gram-negative)* and some non-*Enterobacteriaceae* species (*Pseudomonas aeruginosa* and *Acinetobacter* spp.)	Sepsis in neonates Case- control study (retrospective) Tertiary hospital Healthcare/ hospital	Healthcare costs, length of stay, mortality (odds ratio) Stepwise calculation
[49]	Uematsu et al. 2018	Japan, Multi-centre Resistant: 7188 Susceptible: 7717	MRSA	MSSA	Multiple sites Cohort study (retrospective) Acute care hospitals Healthcare/ hospital	Healthcare costs, length of stay, mortality rate Regression
[66]	Jia et al. 2019	China, Multi-centre, Resistant: 331 Susceptible: 331	All types of HAI infections caused by the specified MDR organisms: MRSA, methicillin- resistant Staphylococcus epidermidis (MRSE), vancomycin resistant Enterococcus (VRE), extended-spectrum B-lactamases producing (ESBLs) Escherichia coli and Klebsiella pneumoniae, carbapenem-resistant Escherichia coli (CR-E. coli) and Klebsiella pneumoniae (CR-Kp), carbapenem- resistant Acinetobacter baumannii (CR-AB), and carbapenem-resistant Pseudomonas aeruginosa (CR-PA)	Healthcare-associated Infection (HAI), caused by the specified non- MDR organisms:: MRSA, methicillin- resistant Staphylococcus epidermidis (MRSE), vancomycin resistant Enterococcus (VRE), extended-spectrum B-lactamases producing (ESBLs) Escherichia coli and Klebsiella pneumoniae, carbapenem-resistant Escherichia coli (CR-E. coli) and Klebsiella pneumoniae (CR-Kp), carbapenem- resistant Acinetobacter baumannii (CR-AB), and carbapenem-resistant Pseudomonas aeruginosa (CR-PA)	Multiple sites Case- control study Secondary + tertiary hospitals Healthcare/ hospital	Healthcare costs, Significance test
[69]	Zhen et al. 2020	China, Multi-centre, Resistant: 1335 Susceptible: 1397	MRSA	MSSA	Multiple sites Case- control study (retrospective) Tertiary hospital Healthcare/ hospital	Healthcare costs, length of stay, mortality rate Matching
[53]	Zilberberg et al. 2019	USA, Multi-centre Resistant: 1059 Susceptible: 7910	gram-negative organisms: P aeruginosa, A baumannii, Stenotrophomonas maltophilia and Enterobacteriacea	Carbapenem-susceptible gram-negative respiratory organisms: P aeruginosa, A baumannii, Stenotrophomonas maltophilia and Enterobacteriacea	Lung Cohort study (retrospective) Not reported Healthcare/ hospital	Healthcare costs, length of stay, mortality rate, readmission rate Regression
[56]	Francois et al. 2016	France, National level, Resistant: 262 Susceptible: 198	Resistant E. coli	Susceptible wild E. coli	Urinary tract Cross- sectional study Primary care setting Healthcare/ hospital	Healthcare costs Significance test
[48]	Uematsu et al. 2017	Japan, Multi-centre Resistant: 93838 Susceptible: 2181827	MRSA	MSSA	Multiple sites Case- control study (retrospective) Acute care hospitals Payer’s perspective	Healthcare costs, length of stay, mortality rate Descriptive
[61]	Giraldi et al. 2019	Italy, Single centre, Resistant: 122 Susceptible: 122	MRSA Vancomycin-resistant enterococci (VRE), MDR Acinetobacter baumannii (MDRAB), Multidrug-resistant Pseudomonas aeruginosa (MDRPA). Carbapenem-resistant Enterobacteriaceae (CRE). (hospital associated infections)	MSSA Vancomycin-susceptible enterococci (VSE). non-MDR Acinetobacter baumannii (Non-MDRAB), Non-MDR Pseudomonas aeruginosa (Non-MDRPA), Carbapenem-susceptible Enterobacteriaceae (CSE) (hospital associated infections)	Multiple sites Cohort study (retrospective) Tertiary hospital Healthcare/ hospital	Attributable costs, length of stay, mortality rate, Significance test
[55]	Inagaki et al. 2019	USA, National level, Resistant: 44653 Susceptible: 47436	MRSA	MSSA	Blood stream Cohort study (retrospective) Secondary + tertiary hospitals Healthcare/ hospital	Healthcare costs, mortality (odds ratio) Regression
[50]	Tabak et al. 2019b	USA, Multi-centre, Resistant: 523 Susceptible: 1381	MDR P. aeruginosa isolates	Non-MDR P. aeruginosa	Respiratory infections Cohort study (retrospective) Acute care hospitals Healthcare/ hospital	Healthcare costs, length of stay, readmission rate Regression
[47]	Uematsu et al. 2016	Japan, Multi-centre, Resistant: 634 Susceptible: 87427	MRSA	MSSA	Lung (pneumonia) Case- control study (retrospective) Acute care hospitals Healthcare/ hospital	Healthcare costs, length of stay, mortality rate Regression
[54]	Thorpe et al. 2018	USA, National level, Resistant: 123254 Susceptible: 12766374	All types of resistance bacteria	All types of susceptible bacteria	Multiple sites Cross- sectional study (Panel survey) Not reported Payer’s perspective	Healthcare costs Regression
[71]	Zhen et al. 2017	China, Single centre, Resistant: 2126 Susceptible: 854	Carbapenem resistance Acinetobacter baumannii	Carbapenem susceptible Acinetobacter baumannii	Multiple sites Case- control study (retrospective) Tertiary hospital Healthcare/ hospital	Healthcare costs mortality rate, Stepwise calculation
[59]	Mora-Guzman et al., 2020	Spain, Single centre, Resistant: 40 Susceptible: 120	MDR Carbapenemase-producing Enterobacteriaceae (CPE)	Susceptible Enterobacteriaceae	Intra-abdominal infection (IAI) Case- control study Tertiary hospital Healthcare/ hospital	Healthcare costs, length of stay, readmission rate Significance test
[73]	Zhen et al. 2021	China, Multi-centre, Resistant: 3048 Susceptible: 3048	MDR or SDR infections or colonisations caused by Staphylococcus aureus, Enterococcus faecalis, Enterococcus faecium, Escherichia coli, Klebsiella pneumonia, Pseudomonas aeruginosa, and Acinetobacter baumannii	Susceptible infections	Multiple sites Case- control study (retrospective) Tertiary hospital Healthcare/ hospital (also societal for national level cost)	Healthcare costs, length of stay, Descriptive
[74]	Iskandar et al. 2021	Lebanon, Multi-centre, Resistant: 911 Susceptible: 854	Mix of both SDR and MDR	Susceptible infections	Multiple sites Cross- sectional study (prospective) Tertiary hospital Payer’s perspective	Healthcare costs, length of stay Regression
[65]	Nelson et al. 2021	USA, Multi-centre/national level Resistant: 25006 Susceptible: 240350	MDR infections by MRSA, VRE, ESBL, CRE, CR Acinetobacter, MDR Pseudomonas	Non- MDR infections	Multiple sites Cohort study (retrospective) Not reported Healthcare/ hospital	Attributable costs, Regression, multivariable LGM

Majority of the included studies (n = 18, 62.1%) were conducted in multiple centres [47–50, 51, 53–57, 65, 66, 69, 73–76], 48.3% studies (n = 14) were about gram negative bacteria [50, 51, 53, 56–60, 62, 68, 70–72, 75], 27.6% (n = 8) were about gram positive bacteria [47–49, 55, 63, 67, 69, 76] and rest (n = 7, 24.1%) were a mix of both [54, 61, 64–66, 73, 74]. Out of the total included studies, 27.6% (n = 8) focused on hospital acquired infections (HAI) [50, 53, 59–61, 63, 66, 72], 10.3% (n = 3) on community acquired infections (CAI) [47, 51, 56], and 24.1% (n = 7) included both HAI and CAI [49, 57, 64, 65, 67, 74, 75]. The remainder of studies did not clearly distinguish type of infection. The majority of the studies (n = 26, 89.6%) were conducted from hospital or healthcare perspective [47, 49, 50, 51, 53, 55–71, 73, 75, 76] and only 10.3% studies (n = 3) were conducted from patients/payer’s perspective [48, 54, 74]. Out of 29 studies, 44.8% (n = 13) were conducted in tertiary care settings [58–63, 68–72], 13.8% (n = 4) were conducted in mix of secondary and tertiary care settings [55, 64, 66, 76] and 3.4% (n = 1) each were conducted in primary [56] and secondary [57] care settings.

**Fig 2:**
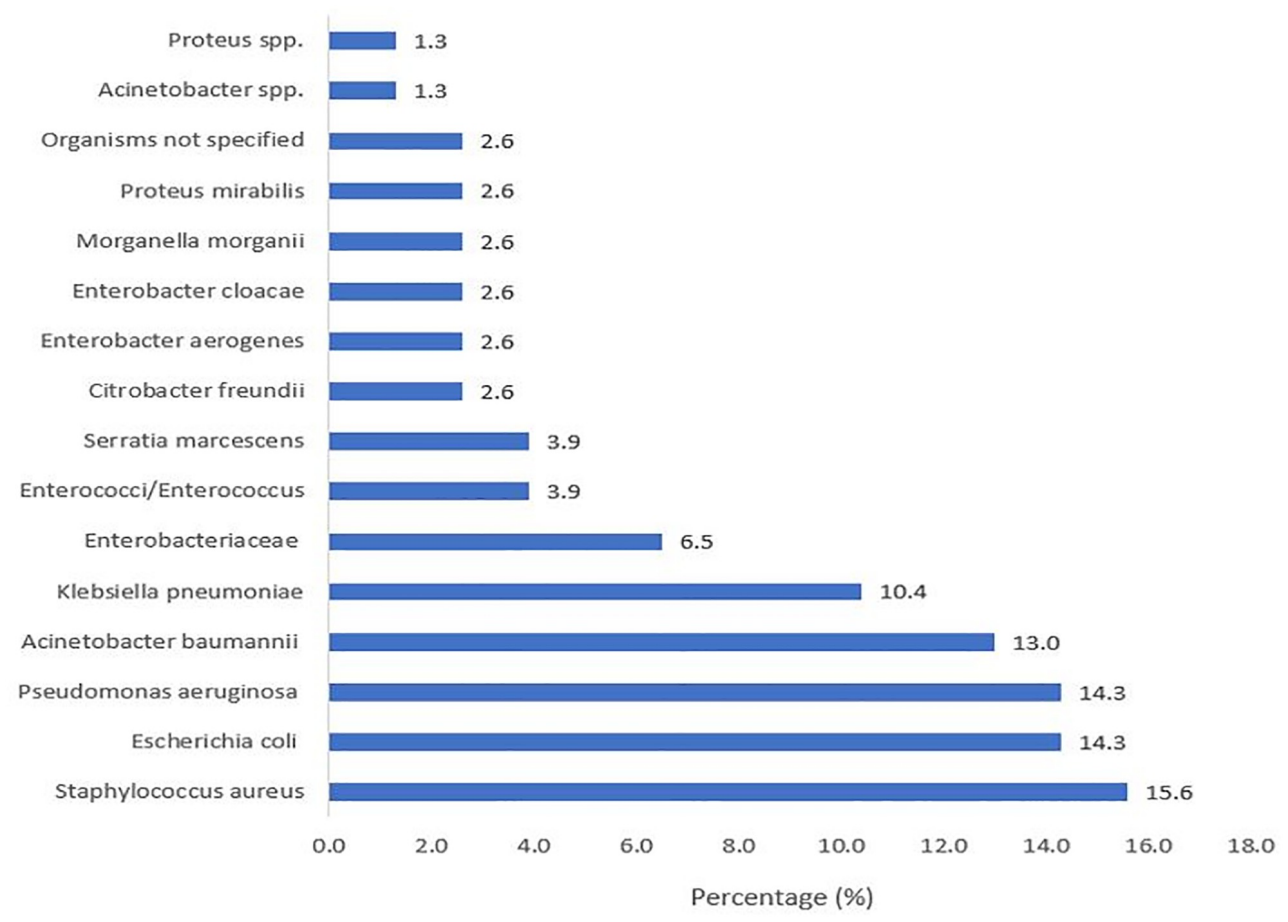
The most studied resistant bacteria reported in the reviewed studies.

Out of total studied bacteria (n = 77, ≥ 1 resistant bacteria studied in one study), the most studied species were *Staphylococcus aureus* (n = 12, 15.6%) [47–49, 55, 61, 64–66, 69, 73, 74, 76], *Escherichia coli* (n = 11, 14.3%) [51, 56, 57, 62, 64–66, 72–75], *Pseudomonas aeruginosa* (n = 11, 14.3%) [50, 51, 53, 58, 61, 65, 66, 68, 73–75], *Acinetobacter baumanii* (n = 10, 13.0%) [51, 53, 61, 65, 66, 71, 73–75] and *Klebsiella pneumonia* (n = 8, 10.4%) [51, 62, 64–66, 70, 73, 75]. Details are presented in Fig 2.

### Methods used to estimate the burden of antibiotic resistance at patient level

When estimating the burden of antibiotic resistance, regression analysis (n = 16, 55.2%) was the most used method [47, 49, 50, 51, 53–55, 58, 60, 63, 65, 70, 72, 74, 75] followed by significant tests (n = 40, 13.8%) [56, 59, 61, 66] and matching (n = 3, 10.3%) [62, 69, 76]. Other methods used in analysis were stepwise calculation (n = 2, 6.9%) and economic [57] and multistate [64] modelling (n = 1, 3.4% each). Although some of the studies reported regression analysis along with general linear model (GLM) or generalised linear mixed model (GLMM) in their method, regression was the main analysis method in their studies. Details are summarised in Table 3.

**Table 3:** Analysis methods and perspectives used to estimate the burden of antibiotic resistance.

Methods of analysis	Hospital/healthcare perspectives % (n = 26)	Payer’s perspectives (n = 3)	Total (n = 29)
Regression analysis	48.3%	6.9%	55.2%
Significance tests	13.8%	0%	13.8%
Matching	10.3%	0%	10.3%
Stepwise calculation	6.9%	0%	6.9%
Multistate model	3.4%	0%	3.4%
Economic model	3.4%	0%	3.4%
Survival analysis	3.4%	0%	3.4%
Other (e.g., descriptive)	3.4%	3.4%	6.9%

### Quality of included studies

To assess the methodological quality of studies, we checked the study design and used suitable quality assessment tools. Our review included 15 cohort studies, nine case-control studies, three cross-sectional [54, 56, 74] and two modelling studies [57, 64]. In addition to those stated in our protocol (i.e. Newcastle and Ottawa Scale, Philips Scale), we used the AXIS appraisal tool for cross-sectional studies [34]. Based on standardised scoring across tools, the highest possible quality score of a study in this review is one (1) and the lowest possible score is zero (0), where high score (≥0.76) means low risk of bias, score between 0.51 and 0.75 means medium risk of bias and from zero (0) to 0.50 means high risk of bias. Quality scores of included studies in this review ranged from 0.51 to 0.89 and the median quality score of the overall 29 studies were 0.79 with an interquartile range (IQR) of 0.67–0.79. Out of 29 studies, 19 studies (65.5%) had quality scores ≥0.76 (low risk of bias), 10 studies (34.5%) had quality scores between 0.51 and 0.75 (medium risk of bias) and there were no studies which had quality score 0.5 or less. Details on scores of individual studies are reported in the S1 Table.

From the quality assessment scores reported in Table 4 below, we can assume that uncertainties and risk of bias were considered seriously in studies of the economic burden of ABR published since January 2016: the risk of bias scores are better in our study than in previous review studies [27, 28]. However, there were still some methodological limitations as the scores did not cross above 0.89 and the minimum score was 0.51.

**Table 4:** Quality of the included studies published between January 2016 and December 2021.

S.N.	Description of studies	Median	Interquartile		Interquartile range (IQR)
			Q1	Q3	
**A.**	**Study types**				
1.	Cohort studies (n = 15)	0.79	0.73	0.79	0.06
2.	Case-control studies (n = 9)	0.79	0.68	0.89	0.21
3.	Cross-sectional studies (n = 3)	0.79	0.74	0.79	0.05
4.	Modelling studies (n = 2)	0.56	0.54	0.59	0.05
**B**	**Overall studies (n = 29)**	0.79	0.67	0.79	0.12

### Attributable costs of antibiotics resistance

As there were considerable variations in method of estimation of healthcare cost of resistant and susceptible infections, and high level of heterogeneity in the healthcare cost (I^2^ >90% even in sub-group analysis), we conducted narrative synthesis. From the review of 29 studies, we found that attributable cost (adjusted mean, adjusted based on the World Bank deflation and exchange rate for 2020) of resistant infections ranged from minus (-) US$ 2,371.4 (p = 0.045, higher for MSSA infections compared to MRSA infections) to US$ 29,289.1 (p = 0.03, higher for Vancomycin-resistant enterococci (VRE) infections than VSE infections). Out of 29 studies, 62% studies (n = 18) clearly reported significantly higher attributable costs for resistant infections compared to susceptible infections. However, three out of 29 studies (10.3%) had higher healthcare cost for susceptible infections compared to resistant infections, although among these only one study (3.4%) reported significantly higher healthcare costs for susceptible infection than resistant infection. Detail about the overall costs of resistant infections, susceptible infections and attributable costs along with p values are reported in the S2 Table.

Out of 29 reviewed studies, 89.6% reported costs from hospital or healthcare perspective, where the attributable costs for resistance infections compared to susceptible infections, ranged from negative (-) US$ 2,371.4 to US$ 29,289.1. A small number of studies (n = 3, 10.3%) reported costs from patient or payer’s perspective; in these studies, the cost ranged from US$ 2,524.9 to US$ 23,922.5, and the cost of resistance infection in two out of three studies were significantly higher for resistant infections at 95%, p<0.05. Details are provided in S3 Table.

Only 65.5% studies (19 out of 29) clearly reported healthcare settings: primary care (n = 1), secondary care (n = 1), tertiary care (n = 13) and mixed of secondary and tertiary (n = 4). While comparing the attributable costs of antibiotic resistance to susceptible infection, based on healthcare settings, it was reported that primary healthcare had the attributable cost of minus (-) US$ 9.0 (p = 0.63), meaning the healthcare cost for resistant and susceptible infections were almost similar at primary care setting. However, at secondary care setting, the attributable cost (adjusted) was US$ 337.7 (p value not reported), meaning the cost for resistant infection was slightly higher than susceptible infection. Likewise, the healthcare cost for resistant infections were considerably higher at tertiary care settings as the attributable costs ranged from US$ 2,291.4 to US$ 29,289.1 (attributable costs in 10 studies out of 13 reported significantly higher healthcare costs for resistant infections with 95% CI, p<0.05). The attributable costs at mixed (secondary and tertiary) settings varied considerably (minimum—US$ 2,371.4, p = 0.045; maximum US$ 4,300.7, p < 0.01). Details about the costs are reported in the S4 Table.

Out of 29 included studies, 17 were cohort (including two modelling studies), nine case-control and three cross-sectional studies. While comparing the attributable costs of antibiotic resistance (adjusted) based on study design, it ranged from (minus) -US$ 2,371.4 (p = 0.045) to US$ 28,491.3 (p<0.001) in cohort studies. The attributable cost in case—control studies ranged from US$ 2,291.4 (p = 0.01) to US$ 29,289.1 (p = 0.03). The cost in cross- sectional studies ranged from (minus)–US$ 9.0 to US$ 2,834.2. Details about the attributable costs including adjusted healthcare costs for resistant infections and susceptible infections by study type are reported in the S4 Table.

Out of 29 studies, 21 studies (72.4%) were from High Income Economies (HIE) and rest of the studies (27.6%) were from Upper-Middle-Income Economies (UMIE). While looking at the attributable healthcare costs due to ABR in HIE, it ranged from negative (-) US$ 2,371.4 (means- susceptible group had higher cost than ABR group) to US$ 29,289.1 per resistance case (12 out of 21 studies reported significantly higher for resistant infections at 95% CI, p<0.05) and in UMIE it ranged from US$ 2,291.4 to US$ 12,043.5 per case (7 out of 8 studies from UMIE reported significantly higher for resistant infection, at 95% CI, p<0.05). All the negative attributable costs in three studies were identified in HIE (two in the USA and one in France) but not in UMIE. Details are provided in S6 Table.

### Segregated costs for resistant and susceptible infection

Seven studies out of 29 reported segregated costs for resistant and susceptible infections. Out of seven, five studies were from China, one from the United States and one from Lebanon. These studies reported segregated cost on medicine, antibiotics, diagnostic test, bed, materials and so on (details are reported in S7 Table). In six out of seven studies, among all cost items, the highest cost (adjusted) was reported on drugs for both resistant (US$ 13,070.4) and susceptible infections (US$ 8,064.4) by Zhen et al. (2017) [71]. Likewise, the highest attributable cost (adjusted) on drugs was reported as US$ 6,157 by Zhen et al. (2020) [69]. Details about the segregated costs for resistant infection, susceptible infection and attributable costs are reported in S7 Table.

### National level costs of resistance infections

Out of 29 studies, five studies provided extrapolated costs of resistant infections at a national level of the respective country. The highest burden of antibiotic resistance infections at a national level was found to be in China (77 billion US$ from a societal perspective: 35 billion from direct costs and 42 billion of indirect costs) [73], followed by the USA due to MDR bacteria for a year ($4.6 billion) [65] and Japan due to MRSA (US$ 2 billion) [48]. Details are provided in Table 5.

**Table 5:** Costs at national level due to antibiotic resistant infections.

Ref no.	First author and date of publication	Study country	Bacteria and site of infections	Method	Costs at national level*
[57]	Naylor et al. 2019 (studied during 2011–2012)	England, UK	ABR E. coli, Blood stream	Multistate models, Extrapolation	They estimated an annual excess cost of over 14 million pounds in 2012 for E. coli bacteraemia and over £500,000 resistance to at least one tested antibiotic.
[48]	Uematsu et al. 2017 (studied during 2014–2015)	Japan	MRSA, Multiple sites	Descriptive, Extrapolation	Total incremental burden of MRSA was estimated to be US $2 billion (3.41% of total hospitalization costs).
[54]	Thorpe et al. 2018 (Studied during 2002–2014)	USA	All types of resistance bacterial infections, Multiple sites	Regression, Extrapolation	They estimated the annual incremental treatment cost as $1,383 per infection. Based on the number of cases in 2014, this amounts to a national treatment cost of approximately $2.2 billion per year.
[73]	Zhen et al. 2021 (studied during 2013–2015)	China	Multi-drug resistant and single drug resistant bacteria Multiple sites	Descriptive, Extrapolation	Total societal economic cost attributed to antibiotic resistance in inpatients in China of $77 billion (95% UI $67 billion–$87 billion), including $35 billion (95% UI $32 billion–$38 billion) of direct cost and $42 billion (95% UI $35 billion–$49 billion) of indirect cost. The attributable total economic cost is equivalent to 0.37% of China’s GDP in 2017, among which, $20 billion (95% UI $16 billion–$24 billion) was caused by SDR infection or colonisation, and $ 57 billion (95% UI $ 51 billion–$ 63 billion) by MDR infection or colonisation.
[65]	Nelson et al. 2021 (studied during 2007–2015)	USA	Multi-drug resistant bacteria Multiple sites	Multivariate LGM, Extrapolation	We estimate that infections due to these pathogens resulted in $4.6 billion (95% CI, $4.1–$5.1 billion) during this 1-year period. Aggregate community-onset positive cultures ($2.7 billion; 95% CI, $2.3–$3.2 billion) accounted for higher total cost than those with onset in the hospital ($1.9 billion; 95% CI, $1.7–$2.1 billion). Similarly, non-invasive infections ($2.8 billion; 95% CI, $2.4–$3.3 billion) accounted for higher total cost than invasive infections ($1.8 billion; 95% CI, $1.6–$2.0 billion). The pathogens with the highest aggregate costs were MRSA with $1.2 billion (95% CI, $0.9–$1.4 billion) for community-onset infections and $580.2 million (95% CI, $459.8–$700.5 million) for hospital-onset infections and ESBL with $752.4 million (95% CI, $431.9–$1073.0 million) for community-onset infections and $470.5 million (95% CI, $339.8–$601.2 million) for hospital-onset infections.

* The costs reported in the table were presented in their original state (i.e., no adjustments were made)

### Length of stay

Out of 29 studies, 21 studies provided information about the length of stay (LoS) to enable estimation of the mean (weighted) length of stay for resistant and susceptible infections. Out of these 21 studies, two studies did not provide IQR or SD, therefore only 19 studies were included for Meta-Analysis. We also estimated the mean length of stay by study design, study perspectives, healthcare settings and income category of countries.

**Fig 3:**
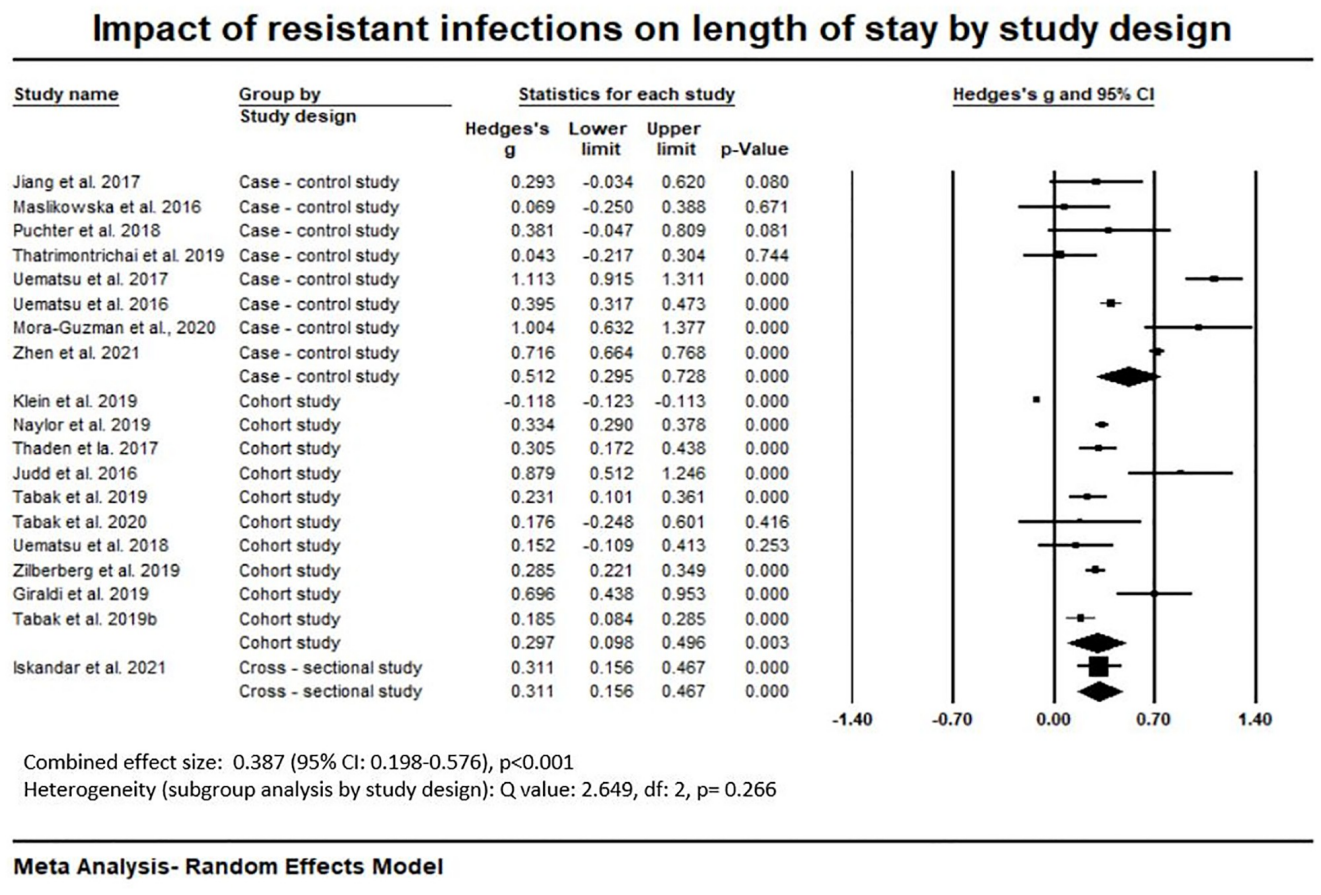
Impact of resistant infections on length of stay at hospital by study design.

Our Meta-Analysis of LoS (S1 Fig) shows that the combined effect size in resistant infection is higher than in susceptible infection (Hedges’s g effect size: 0.387, 95% confidence interval: 0.198–576, p < 0.001). Out of 19 eligible studies, 68.4% studies (n = 12) showed significantly higher impact of resistant infections on LoS compared susceptible infections (S8 Table). Sub-group analysis by study design (Fig 3) shows that combined effect size in resistant infection compared to susceptible infection in case-control studies (Hedges’s g: 0.512, 95% CI 0.295–0.728, p < 0.001) was highest compared to cross-sectional study (Hedges’s g: 0.311, 95% CI 0.156–0.467, p <0.001) (there was only one study in this design) and cohort studies (Hedges’s g: 0.297, 95% CI 0.098–0.496, p = 0.003). Considering between-study heterogeneity, the Q value was small and p value was ≥ 0.05, (Q value: 2.649, df: 2, p = 0.266), indicating there was low chance of between-study heterogeneity within these groups (Fig 3).

The effect size on LOS was higher in studies that measured from the payer’s perspective (Hedges’s g: 0.710, 95% CI -0.076–1.495, p = 0.077) compared to healthcare or hospital perspective (Hedges’s g: 0.347, 95% CI 0.151–0.544, p = 0.001) (S2 Fig). Combined effect size was highest in a tertiary hospital setting (Hedges’s g: 0.480, 95% CI 0.274–0.686, p <0.001), followed by in secondary care setting (Hedges’s g: 0.334, 95% CI 0.290–0.378, p < 0.001). Details are reported in S3 Fig. The combined effect size on LoS was slightly higher in high income economies (HIE) (Hedges’s g: 0.393, 95% CI 0.213–0.574, p < 0.001) compared to upper- middle income economies (UMIE) (Hedges’s g: 0.355, 95% CI– 0.021–0.690, p = 0.037) (S4 Fig).

Table 6 shows the weighted mean of LoS (n = 21) for resistant infection as 26.4 days (95% CI: 19.9 days—32.8 days), whereas the LoS was considerably lower for susceptible infection (weighted mean 18.9, 95% CI: 14.5 days– 23.4 days). Likewise, the weighted mean of attributable LoS to resistant infection was 7.4 days (95% CI: 3.4 days– 11.4 days). We also estimated weighted mean of the attributable LoS by study design (n = 21), healthcare setting (n = 12), study perspective (n = 21) and income category of the country (n = 21). We found that the case-control studies reported the highest attributable LoS (10.4 days), and cross-sectional study (only one study) reported the lowest attributable LoS (2.1 days) among study designs (i.e., case-control, cohort studies and cross-sectional) (Table 5). Likewise, attributable LoS was higher at a tertiary hospital (7.1 days, 95% CI: 3.3 days– 10.9 days) compared to secondary care setting (3.3 days, CI not available). Mean LoS from payer’s perspective was considerably higher (20.5 days) compared to the mean LoS from a healthcare or hospital perspective (6 days, 95% CI: 3.6 days– 8.4 days). No data was available from studies that took a societal perspective. Table 5 also shows that attributable LoS in HIE (8.4 days, 95% CI: 2.7days—13.9 days) was considerably higher than that in UMIE (5.1 days, 95% CI: 2.3 days– 7.8 days). Details about the length of stay estimates for resistant and susceptible infections by study design, healthcare setting, study perspective and income categories of the countries are presented in Table 6.

**Table 6:** Comparisons of length of stay of resistant and susceptible infections.

SN	Description of the variables		Mean LoS (days)*	95% confidence	
				Lower bound (days)	Upper bound (days)
**1.**	**Excess length of stay**				
	**By study design**				
	Case—Control Study (n = 10)		10.4	2.6	18.3
	Cohort Study (n = 10)		4.9	1.1	8.7
	Cross—Sectional Study (n = 1)		2.1	-	-
	**By healthcare setting**				
	Tertiary hospital (n = 11)		7.1	3.3	10.9
	Secondary + Tertiary hospitals (n = 1) **		-0.7	-	-
	Secondary hospitals (n = 1) **		3.3	-	-
	**By income categories**				
	High income economies (HIE) (n = 15)		8.4	2.7	13.9
	Upper-middle-income economies (UMIE) (n = 6)		5.1	2.3	7.8
	**By study perspective**				
	Healthcare/Hospital (n = 19)		6.0	3.6	8.4
	Payer’s perspective (n = 2) **		20.5	-	-
**2.**	**Overall length of stay**				
	Resistant infection (n = 21)		26.4	19.9	32.8
	Susceptible infection (n = 21)		18.9	14.5	23.4
	Excess length of stay (n = 21)		7.4	3.4	11.4
**3.**	**Length of stay for resistant and susceptible infections**				
	**By study design**				
	Case-control study (n = 10)	Resistant infection	34.0	25.0	43.0
		Susceptible infection	23.6	17.1	30.1
	Cohort study (n = 10)	Resistant infection	20.5	11.8	29.1
		Susceptible infection	15.5	9.4	21.6
	Cross- sectional study (n = 1)	Resistant infection	8.8	-	-
		Susceptible infection	6.7	-	-
	**Healthcare Setting (resistant infection)**				
	Tertiary hospital (n = 11)	Resistant infection	27.3	18.3	36.3
		Susceptible infection	20.2	12.6	27.8
	Secondary + Tertiary hospitals (n = 1)	Resistant infection	7.7	-	-
		Susceptible infection	8.4	-	-
	Secondary hospitals (n = 1) **	Resistant infection	10.9	-	-
		Susceptible infection	7.6	-	-
	**By income categories**				
	High income economies (HIE) (n = 15)	Resistant infection	25.2	16.9	33.4
		Susceptible infection	16.8	12.6	21.1
	Upper-middle-income economies (UMIE) (n = 6)	Resistant infection	29.3	16.0	42.6
		Susceptible infection	24.2	10.6	37.8
	**By study perspective**				
	Hospital/Healthcare (n = 19)	Resistant infection	25.6	19.8	31.5
		Susceptible infection	19.6	14.8	24.3
	Payer’s perspective (n = 2)	Resistant infection	33.5	-	-
		Susceptible infection	12.8	-	-

* Weighted means of LoS were calculated using random weight (relative weight) of each study.

**Cannot calculate 95% confidence interval (lower and upper bounds) in these groups as there were only limited number of studies (one or two) available.

### Mortality

Seven studies reported mortality odds ratios along with upper and lower bounds at 95% confidence intervals; 13 studies reported mortality rates for both resistant as well as susceptible infections. Thus, we included 20 studies in the Meta-Analysis of mortality in this review. Therefore, we presented the combined effect size of mortality in odds ratio and also by sub-group analysis (by study design (n = 20), study perspective (n = 20), income category of the country (n = 20) and by healthcare setting (n = 13)). Out of 29, 13 studies reported mortality in percentages (S9 Table), therefore, we reported the overall mean (weighted) mortality rate using random weight (relative weight) calculated from the Meta-Analysis.

**Fig 4:**
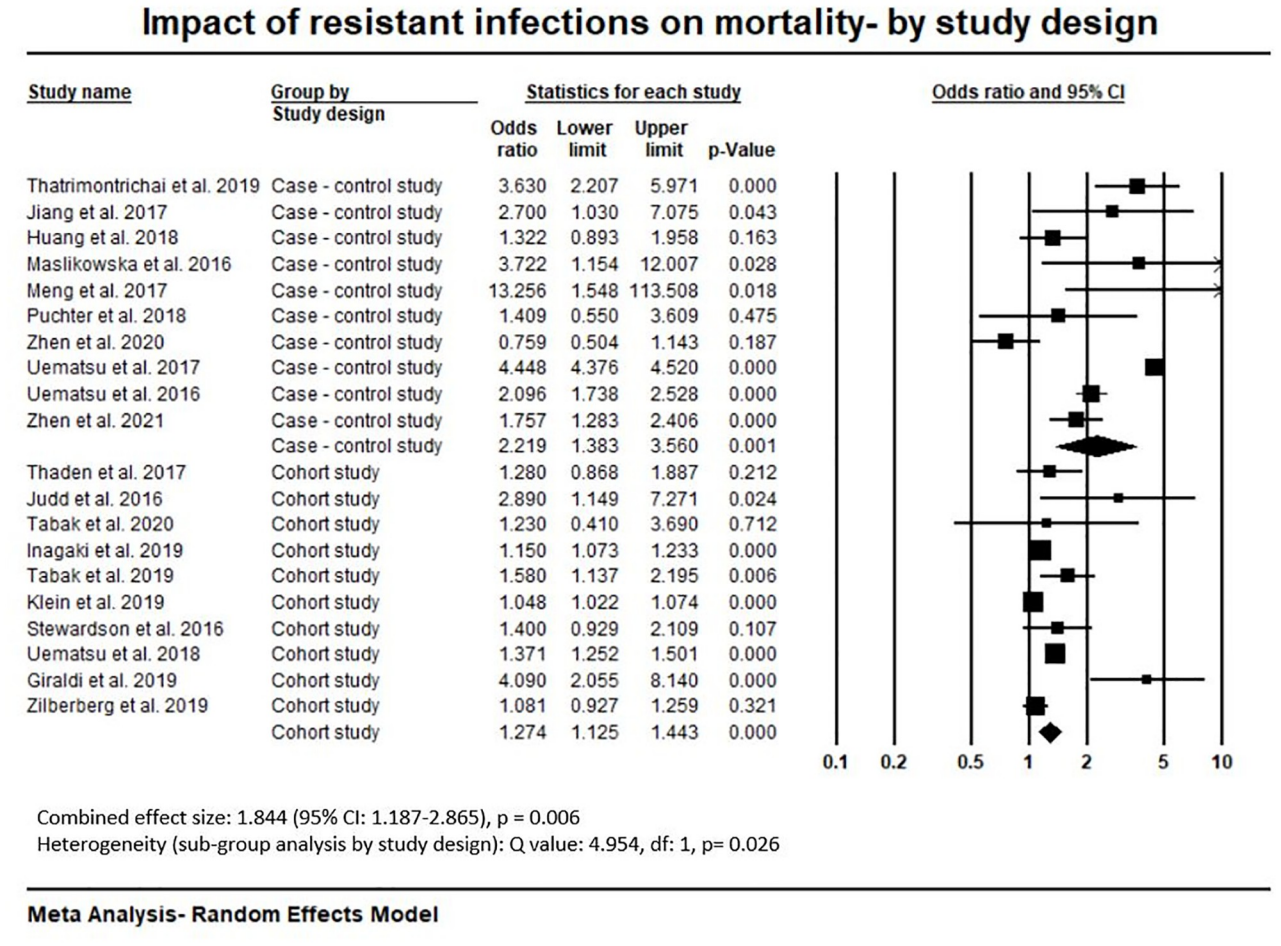
Impact of resistant infections on mortality.

The Meta-Analysis of mortality (S5 Fig) shows the combined odds ratio of 1.844 (95% CI: 1.187–2.865, p = 0.006). This means a patient with a resistant infection had an 84.4% more chance of dying compared to a patient with a susceptible infection. Mortality Meta-Analysis by study design (Fig 4) shows higher mortality odds ratio reported in case-control studies (2.219, 95% CI: 1.383–3.560, p = 0.001) compared to cohort studies (1.274, 95% CI: 1.125–1.443, p< 0.001). Mortality odds ratio was higher in studies that considered payer’s perspective (4.448, 95% CI: 4.376–4.520, p< 0.001) compared to healthcare/hospital perspective (1.481, 95% CI: 1.292–1.699, p< 0.001) (S6 Fig). Mortality data were provided from only two healthcare settings- tertiary, and a mix of secondary and tertiary hospitals. The sub-group analysis of mortality by healthcare setting (S7 Fig) shows higher odds of mortality in tertiary hospitals (1.954, 95% CI: 1.333–2.863, p = 0.001). Higher mortality odds were found in UMIE (1.867, 95% CI 1.107–3.149, p = 1.019) compared to HIE countries (1.764, 95% CI: 1.046–2.974, p = 0.033) (S8 Fig). Mean (weighted) mortality rates for resistant and susceptible infections were calculated. The result shows that mean (weighted) mortality % of resistant infection was higher (17.4%, 95% CI: 10.8%—24.0%) compared to susceptible infection (10.5%, 95% CI: 5.1%—16.0%). Mean excess (weighted) mortality was 6.9% (95% CI: 2.8%—10.9%). Details are presented in S10 Table.

### Readmission

Out of 29 studies, only six studies provided information on readmission of patients with resistant and susceptible infections, and these were in different formats (%, odds ratios and hazards ratio). Three studies provided information about the rate of readmission of patients from each group admitted in a specified period (e.g., in 30 days after discharge), two studies provided odds ratios of readmission and one study provided hazard ratio of readmission. A total of five studies were included in the Meta-Analysis for readmission excluding the study reporting hazard ratios. As the number of studies was very small, we did not conduct a sub-group analysis for this variable. However, we estimated the mean (weighted) readmission rate for resistant, susceptible and excess readmission from the eligible studies.

**Fig 5:**
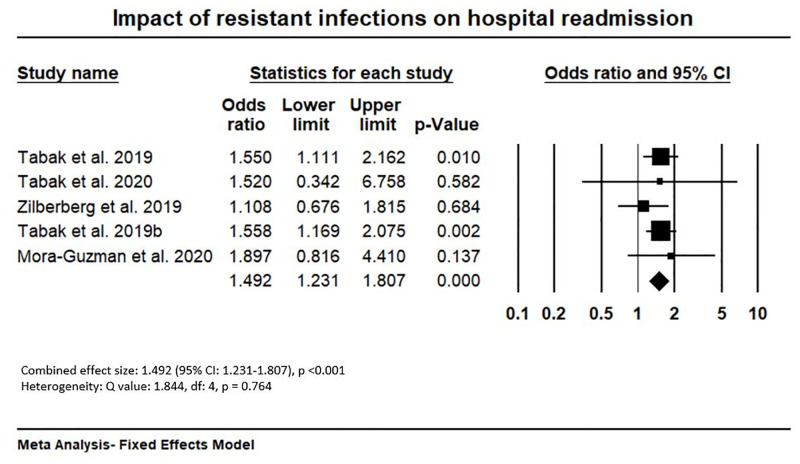
Impact of resistant infections on hospital readmission.

As there were no heterogeneity among studies for readmission rate (Q value: 1.844, df: 4, p: 0.764, I^2^: 0.000), we conducted Fixed Effects Meta-Analysis. Meta-Analysis of readmission (Fig 5) shows a combined odds ratio of 1.492 (5% CI 1.231–1.807, p< 0.001). This means the odds of readmission of patients with resistant infection was 49.2% higher compared to patients with susceptible infection. The lower confidence interval is greater than one (i.e., 1.231). This means the true effect size in similar studies will generally be higher than one, therefore, there will be a higher chance of readmission in resistant infection compared to susceptible infection. As there were limited number of studies (n = 5) on readmission, we did not conduct a sub-group analysis.

Among three studies which reported hospital readmission rate (%), the highest readmission rate (weighted) was reported by Tabak et al. (2019b) in Japan (33.5%) [50] and lowest was reported by Mora-Guzman et al. (2020) in Spain (6.6%) [59]. Highest excess readmission for resistant infection was reported in Spain (10.8%, p = 0.223) and lowest was reported in the USA (1.5%, p = 748) (Details are in S11 Table). We found the mean (weighted) readmission rates (%) for resistant, susceptible, and attributable to resistant infection as 18.8%, 14.1% and 4.7% respectively (Details are in S12 Table).

### Publication bias

**Fig 6:**
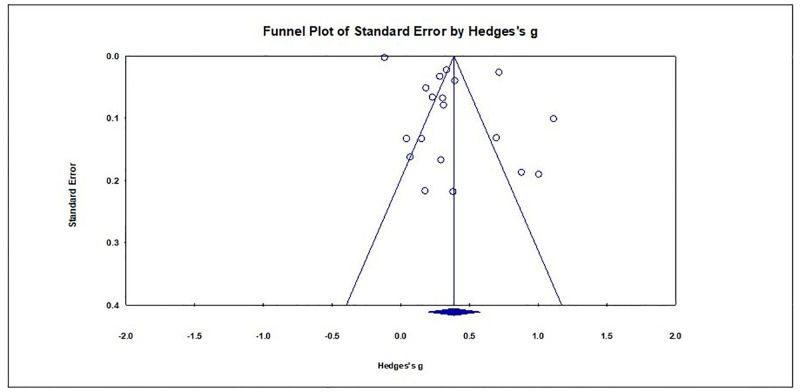
Funnel plot for visual assessment of publication bias.

In this review, publication bias was assessed visually and statistically using a Funnel plot (Fig 6), and Duval and Tweedie’s Trim and Fill method, respectively. Visual inspection shows the plot to be roughly symmetrical but on closer inspection slightly higher number of studies are observed on left hand side. Application of Duval and Tweedie’s Trim and Fill method (S9 Fig) suggested two studies were missing on right hand side of the mean effect line (Hedges’s g: 0.387, 95% CI: 0.198–0.576). ‘Filling in’ these two studies increased the overall effect size slightly (Hedges’s g: 0.440, 95% CI: 0.112–0.768). Therefore, no significant publication bias was observed.

### Economic burden of antibiotic resistance from environmental perspective

Although we intended to assess the economic burden of ABR from an environmental perspective (please see PROSPERO registration CRD42020193886 for the protocol), our search did not identify any original studies taking an environmental perspective. However, we found one narrative review which aimed to estimate the worldwide economic impact of ABR from an environmental perspective [77]. This study, however, used economic burden estimates from studies on humans, due to the lack of original studies conducted assessing attributable costs for ABR in livestock, poultry and environment.

## Discussion

This review evidenced that a limited number of studies have been conducted on the economic burden of antibiotic resistance in recent years, as only 29 studies were identified published since January 2016 that included healthcare costs with suitable comparators (i.e., antibiotic resistant vs susceptible infections). Of these, a very limited number of studies were conducted in primary and secondary care settings. MRSA, E. coli and P. aeruginosa were the top three most studied resistant bacteria in recent economic burden studies. There were variations in the study methodologies, as reported in previous reviews [27, 28]: most of the studies utilised patient record data and the majority of them were retrospective studies [57, 62, 70, 76]. There were wide variations in methodology in estimating the burden and outcomes of the resistant infections, especially in relation to healthcare costs. The healthcare costs of resistant infections found to be considerably higher than susceptible infections and cost varies between healthcare settings. The hospital or healthcare perspective were found to be the most common in economic burden studies compared to payer and societal perspective. China was found to be the country with the highest level of economic burden due to antibiotic resistant. Similar to healthcare cost, hospital length of stay, mortality and hospital readmissions found to be substantially higher for patients with resistant infections compared with susceptible infections.

Overall, 62% of the studies that reported costs show significantly higher attributable costs for resistant infections. It is clear from this review that the healthcare costs of resistant infections are considerably higher than susceptible infections. Cost varies between settings: this review found healthcare costs to be almost equal for resistant and susceptible infections in primary care setting [56], slightly higher for resistant infections in secondary care setting [57] and considerably higher in tertiary care settings [58, 60, 62, 63, 68, 70, 71].

A previous review reported the attributable cost for resistant infections as ranging from less than US$ 5 to US$ 55,000 per patient episode [22]. In contrast our review found the attributable cost to range from negative (-) US$ 2,371.4 [76] to US$ 29,289.1 per patient episode [63]. In HIE settings there are higher variations in attributable costs of resistant infections, ranging from negative (e.g. a study by Klein et al. 2019 in the USA) [76] to the highest level of attributable costs reported in Germany [63]. However, in the UMIE settings such as China, Lebanon and Thailand, the attributable costs were found to be lower, and the cost range narrower, compared to HIE. The reasons of the lower costs in UMIE may be due to less advanced treatment facilities and lower per unit cost of resources used, but could also reflect differences in exchange rate [42]. Most of the published studies reporting economic burden considered the hospital or healthcare perspective, and reported slightly higher costs [58, 63] than those taking the patients or payers perspective [48].

All negative attributable costs (i.e., higher care costs for susceptible infections) were reported from HIE (such as from the USA and France) [55, 56, 76], but not from UMIE. Out of three studies (10.3%) having higher cost for susceptible infections, only one (3.4%) was significantly higher compared to resistant infection (attributable cost -US$ 2371.4, p = 0.045, MRSA vs MSSA, in the USA) [76]. Significantly higher costs for the susceptible infections compared to resistant in this study may be due to several reasons, such as their results were based on diagnostic billing codes [76], which may reflect bias in reporting and billing for MRSA infections and There may have problems in estimating true costs using data for other purposes [51, 57, 71].

At the national level burden of antibiotic resistance estimated through extrapolation of results reported in a number of studies [48, 54, 57, 65, 73], a study in China reported the highest attributable costs due to MDR bacteria [73] followed by the USA due to the same types of bacteria [65]. The higher burden of resistant infections in China maybe because their estimated resistant cases were 12,098,752 (5,113,276 SDR & 6,985,476 MDR) [73], which is 10 times higher than estimated resistant cases (1.2 million) in the USA [54].

Similar to healthcare costs, mean (weighted) excess LoS found to be higher in resistant infection per patient episode (26.4 days) than the susceptible infection (18.9 days), and excess LoS to resistant infection was 7.4 days. The highest LoS was reported in a study by Uematsu et al. 2017 in Japan (weighted mean 58.1 days) for resistant infections due to MRSA [48]. The highest LoS in this study may be because they included relatively older population in their study who were already vulnerable due to age factor (as mean as was 69.4 years), and also may be due to some degree of selection bias in their study as they did not use lab information to select MRSA cases but used anti-MRSA drug information [48]. Results of Meta-Analysis on LoS evidenced that resistant infection had a higher impact on LoS than susceptible infection (effect size 0.387, 95% CI 0.198–0.576, p<0.001). The majority of the studies (68.4%) showed a significant impact on LoS. In this review, excess LoS was found to be higher in a tertiary care setting (7.2 days) compared to secondary care setting (2.1 days). This may be because of the obvious reason as more severe patients are admitted in tertiary care settings who need weeks or months to recover. Excess LoS was found to be higher in the patients from HIE (8.4 days) and from the payer’s perspective (20.5 days) and highest at case-control study (10.4 days) compared to other respective groups (e.g., LMICs, healthcare perspective, cohort and cross-sectional respectively).

In this review, we found a higher mean (weighted) mortality rate for resistant infection (17.4%) compared to the susceptible infection (10.5%). The highest mean (weighted) mortality rate (39.6%) was reported by Putcher et al. (2018) in Germany [63]. This may be because they included all-cause mortality of a group of high-risk patients who had surgical site infections, bloodstream infection (BSI), intra-abdominal infections and infections of organs within the visceral cavity due to vancomycin-resistant enterococci (VRE) [63]. The lowest mortality rate (weighted) was reported by Zhen et al. (2021) (3.4%) in China [73], this may be because they included all types of drug resistant patients and there was only a small number of patients who underwent surgery or were admitted to ICU (73). Likewise, the excess mortality rate in our review was found to be 6.9%. Meta-Analysis of mortality odds ratios of resistant and susceptible infections also concluded that significantly higher mortality is associated with resistant infection as the combined odds ratio was 1.844 (95% CI: 1.187–2.865, p = 0.006). The pooled estimate of mortality odds ratio reported in already published Meta-Analysis conducted in developing countries reported slightly higher than our review finding (2.828, 95% CI: 2.231–3.584) [6]. This may be because they included studies from developing countries where treatment facilities are less advanced than in developed countries, therefore mortality rates are higher. Out of the total included studies in our Meta-Analysis, 65% of studies (n = 13) reported significantly higher mortality odds compared to resistant infections, 30% of studies reported non-significant but higher mortality rate and only one study reported lower mortality for resistant infection compared to susceptible infection. The only study reporting higher mortality odds ratio for susceptible infection (but not significant i.e. p = 0.187) was reported by Zhen et al. (2020) in China [69]. The reason behind this result may be due to uncertainty in identifying and selecting the patients with resistant or colonized infections, as reported in the study [69].

The mean (weighted) readmission of resistant infection was found to be 18.8% and it was 14.1% for the susceptible infection. The highest readmission rate (weighted mean: 33.5%) was reported in a study by Tabak et al. (2019b) in the USA [50], this may be because they included a group of patients who had a respiratory infection due to multi-drug resistant Pseudomonas aeruginosa [50], which have a relatively higher chance of readmissions than other resistant infections. The lowest readmission rate for resistant infection was reported by Mora-Guzman et al. (2020) in Spain (weighted mean: 6.6%) [59]. This may be because they had a small size (n = 40) which caused uncertainty in results. This review also reported the attributable readmission as 4.7%. Meta-Analysis of odds ratios of the studies also confirmed that resistant infection had significantly higher odds of readmission than susceptible infections (combined odds ratio: 1.492, 95% CI: 1.231–1.807, p<0.001).

This review found a range of variations in the economic and health outcomes in the economic burden studies. For example, healthcare costs for resistant infection (UTI due to E. coli) for the study conducted in a primary care setting in France was almost equal to susceptible infection [cost difference (adjusted): -US$ 9.0, p = 0.6]) [56], where the attributable cost for UTI caused by multiple resistant organisms in the USA in an acute care setting was high (adjusted- US$ 1642.0, (p<0.001) [51]. These variations in outcomes may be due to many reasons, such as the source of data used, study design and methodology used, whether confounding factors adjusted or not, time dependent variables adjusted or not, study setting and study country. Although most of the studies accounted for confounding factors, only two studies included in this review considered time dependent variables using the multistate model [57, 64]. For example, a study by Stewardson et al. (2016) [64] included comorbidities in their analysis as individual covariates and two time varying covariates were considered while patients were at risk for BSI (bloodstream infection)- admission to an intensive care unit (ICU) and surgical procedure. As the costs of healthcare depend on LoS, they also adjusted excess LoS for the baseline covariates before estimating attributable healthcare costs. They performed a Monte Carlo simulation with 10,000 samples to account for parameter uncertainty. Some examples of strong methodologies adopted in estimating costs and LoS considering the onset of resistant infections can be found in a number of studies reviewed in this paper [53, 62, 63, 67]. However, other used total hospitalisation data without considering the onset of infections and estimated costs, LoS and mortality [60]. Regression analysis was mostly used method in assessing the economic burden of antibiotic resistance in this review as reported in a previous review [28]. Among the regression analysis, logistic regression (univariate, multivariate, random intercept, backward), Cox regression analysis, hierarchical generalised regression analysis, gamma regression analysis and linear regression model were generally used.

Although the quality of recent studies on economic burden found to be relatively higher than previous published studies [28] based on quality assessment of the included studies in this review using NOS (for case control, and cohort), Philips (for modelling), AXIS (cross-sectional)—the following recommendations were made for future studies estimating the economic burden of antibiotic resistance:

1. For all studies—clearly report how control groups were identified and selected and how cases and control were matched, and confounding factors were considered, were both cases and control group data collected from the same source, i.e., community or hospitals
2. For cohort studies—we recommend reporting clearly about the follow up of patients (e.g., duration of follow up, % lost in follow up, description of patients who were lost in follow up etc.).
3. For modelling studies—run an alternative version of models to reduce methodological uncertainty and clearly report it, report if any methodological assumptions were made, fix time horizon and report it, assess the quality of the data to be used before using it and report it in the paper.
4. For modelling studies- calibrate the models and report about it, deal with different types of uncertainty and report it, assess competing theories regarding model structure and report it, clearly report about the structural assumptions of the study if made any, conduct half cycle correction and report it.
5. In modelling studies—deal with heterogeneities and repot how these were dealt with (i.e., running the models separately for different sub-groups), test the mathematical logic of the model thoroughly before using and reporting about it.

Although there were many recommendations to conduct the economic burden of resistant infections from a societal perspective [19, 26, 28], no studies were found to be eligible to be included in the analysis. A couple of studies were conducted from a societal perspective along with a healthcare or payer’s perspective, but these were not suitable to be included in our analysis as they did not report economic and health burdens per patient episode [56, 73]. Recent publications show that the burden of ABR is significant and affects every country in the world [1, 2]).

Strengths of this review include a comparison of economic burden in terms of healthcare costs, LoS, mortality and morbidity from healthcare settings, the perspective of the study, study designs and income categories of the countries. This study calculated the adjusted mean of costs length of stay, mortality and readmission for both resistant and susceptible infections. Meta-Analysis of length of stay, mortality and readmission helped us to determine the true effect size of antibiotic resistant infections on these economic and clinical outcomes. Likewise, this review also explored the methodologies in assessing the economic burden of antibiotic resistance, economic and health outcome indicators mostly used in the recent economic burden studies. Moreover, the quality of the studies was assessed, and future recommendations were provided to conduct better quality on the economic burden of ABR. Although there were no eligible studies for review, this review assessed the availability of studies on ABR and livestock, agriculture and environment. We used the PRISMA guidelines to report the review. Due to all of the above reasons, we believe the study and its findings are original, comparative, robust and recent.

### Limitations of the study

This review included only those studies which reported healthcare costs for resistant infections (which was the main focus of the review) along with LoS and health outcomes such as mortality and hospital readmission. Therefore, this review had to exclude a wide range of studies on antibiotic resistant which focussed on health outcomes only. This is the main limitation of this review. Although we aimed to assess the economic burden of ABR from all the above-mentioned aspects, there were limited studies available from primary care setting, and there were no suitable data to account for societal perspectives. Likewise, no studies evaluated the economic burden of ABR from an environmental perspective. Although we found a couple of studies (9 out of 29, 31%) from upper-middle-come economies, such as China and Thailand. We did not obtain any relevant studies on the economic burden of antibiotic resistance from low-income economies (LIE). Therefore, the findings of the review may not be representative of low-income economies. In addition, most of the included studies were retrospective in nature and conducted from a hospital or healthcare perspective. Therefore, there may be methodological bias in capturing all cost components and estimating true costs of antibiotic resistance. We only included articles in English that may have potential language bias. As we searched articles in the databases and depended on the online, this may cause slight publication bias, however assessment of publication bias using Funnel Plot and Trim and Fill showed that there was no considerable publication bias in this review.

## Conclusions

There were a limited number of studies on the economic burden of antibiotic resistance published in recent years which have at least one cost component and suitable comparators. There was a limited number of studies conducted from a societal perspective and at primary care setting in recent years although many recommendations in previous reviews. Although there were variations in the results, recent publications show that health and economic burden of ABR is significant, and it affects every country in the world. The top three most studied resistant bacteria in the economic burden studies were *S*. *aureus*, *E*. *coli and P*. *aeruginosa*. Based on the available evidence from the included studies, attributable costs of resistant infections were generally high leaving some exceptions. Attributable costs of resistant infections were similar to susceptible at primary care settings, slightly higher at secondary care settings and highest at tertiary care settings. Higher attributable costs and variations in the costs were found in HIE compared to UMIE. Similar to attributable costs, excess LoS was highest at tertiary care setting, higher in HIE, higher from patients/payers’ perspective. There were no studies on the economic burden of antibiotics resistance from an environmental perspective, we strongly recommend conducting one in the future.

A range of variations in the economic and health outcomes in the economic burden studies found in this review. Majority of the economic burden studies used regression analysis. Examples of strong methodologies used in estimating costs and LoS considering the onset of resistant infections were also found in a number of studies. The quality of the recent studies was better compared to the studies in the past. However, there was still a risk of bias in assessing the true burden of antibiotic resistant due to some methodological issues. To reduce the methodological caveats, we recommend researchers conduct a prospective study, consider all possible confounding factors, in multicenter, from a different perspective, using enough sample size, in different healthcare settings and use multistate modelling approach whenever possible to demonstrate long-term impacts and to control bias due to time dependent variables.

There were no recent studies that were suitable to assess the burden from a societal perspective and there were no studies covering productivity losses of patients as well as family members due to resistant infections. There were limited studies in primary care settings and no studies from low-income economies (LIE). Therefore, we recommend conducting more economic burden studies from a societal perspective in primary care settings and low-income economies. The findings of the study may be of value to researchers, policymakers, clinicians, and those who are working in the field of ABR control and health promotion across UMIE and HIE. Likewise, the economic and health outcome estimates reported in the study may be useful for future modelling studies to estimate the long-term economic burden of antibiotic resistance.

## Supporting information

S1 Table Quality score of the reviewed studies. (PDF)

S2 Table Healthcare costs of resistant infections, susceptible infections and attributable costs. (PDF)

S3 Table Healthcare costs of resistant infections, susceptible infections and attributable costs by study perspective. (PDF)

S4 Table Healthcare costs of resistant infections, susceptible infections and attributable costs by healthcare settings. (PDF)

S5 Table Healthcare costs of resistant infections, susceptible infections and attributable costs by study design. (PDF)

S6 Table Healthcare costs of resistant infections, susceptible infections and attributable costs by income category of countries. (PDF)

S7 Table Segregated costs for resistant and susceptible infections. (PDF)

S8 Table Length of stay for resistant and susceptible infections. (PDF)

S9 Table Mortality rate and odds ratios for resistant and susceptible infections. (PDF)

S10 Table Comparisons of mean mortality % between resistant and susceptible infections. (PDF)

S11 Table Readmission rate and odds ratios for resistant and susceptible infections. (PDF)

S12 Table Comparisons of readmission % between resistant and susceptible infections. (PDF)

S1 Fig Impact of resistant infections on length of stay at hospital. (PDF)

S2 Fig Impact of resistant infections on length of stay at hospital by study perspective. (PDF)

S3 Fig Impact of resistant infections on length of stay at hospital by healthcare setting. (PDF)

S4 Fig Impact of resistant infections on length of stay at hospital by income category of country. (PDF)

S5 Fig Impact of resistant infections on mortality. (PDF)

S6 Fig Impact of resistant infections on mortality by study perspective. (PDF)

S7 Fig Impact of resistant infections on mortality by healthcare setting. (PDF)

S8 Fig Impact of resistant infections on mortality by income category of country. (PDF)

S9 Fig Duval and Tweedie’s Trim and Fill method for publication bias. (PDF)

S1 Checklist PRISMA checklist 2020 for ‘the economic burden of ABR systematic review & meta-analysis paper’. (PDF)

## Abbreviations

ABR: Antibiotics Resistance
AMR Review: Review on Antimicrobial Resistance
AMR: Antimicrobial Resistance
ART: antiretroviral therapy
CDC: Centre for Disease Control and Prevention
DHSC: Department of Health and Social Care (UK)
HPA: Health Protection Agency
HPS: Health Protection Scotland
ICD: International Classification of Diseases
MDR: multi-drug resistance
NICE: National Institute for Health and Care Excellence
OHID: Office for Health Improvement and Disparities
PHE: Public Health Europe
PHS: Public Health Scotland
PHW: Public Health Wales
PICO: Population, Intervention, Comparison, Outcome
PLHIV: people living with HIV
PRISMA: Preferred Reporting Items for Systematic Review and Meta-Analysis
UKSHA: UK Health Security Agency
WHO: World Health
