# Supplementary material for: The economic burden of antibiotic resistance: A systematic review and meta-analysis
Source: PLoS One. 2023 May 8;18(5):e0285170. doi: 10.1371/journal.pone.0285170 (PMC10166566; doi:10.1371/journal.pone.0285170)
Supplement: S1 Table — (PDF) [file pone.0285170.s001.pdf]

Supplementary Table 1. Quality score of the reviewed studies along with study design and healthcare settings

Study Ref No.	Study design, healthcare settings	Quality score (out of 1)
(70)	Case- control study (retrospective), Tertiary hospital	0.78
(67)	Case- control study, Setting not reported	0.89
(78)	Cohort study (retrospective), Secondary + tertiary hospitals	0.56
(62)	Case- control study (retrospective), Tertiary hospital	0.89
(72)	Case- control study (retrospective), Tertiary hospital	0.89
(57)	Cohort study (retrospective), Secondary healthcare setting	0.51
(63)	Case- control study (retrospective), Tertiary hospital	0.79
(60)	Cohort study (prospective), Tertiary hospital	0.56
(58)	Cohort study (retrospective), Tertiary hospital	0.78
(64)	Cohort study (retrospective), Secondary + tertiary hospitals	0.61
(77)	Cohort study (retrospective), Acute care hospitals	0.79
(76)	Cohort study (retrospective), Acute care hospitals	0.79
(68)	Case- control study (retrospective), Tertiary hospital	0.67
(49)	Cohort study (retrospective), Acute care hospitals	0.79
(66)	Case- control study, Secondary + tertiary hospitals	0.68
(69)	Case- control study (retrospective), Tertiary hospital	0.89
(53)	Cohort study (retrospective), Setting not reported	0.56
(56)	Cross- sectional study, Primary care setting	0.68
(48)	Case- control study (retrospective), Acute care hospitals	0.67
(61)	Cohort study (retrospective), Tertiary hospital	0.79
(55)	Cohort study (retrospective), Secondary + tertiary hospitals	0.79
(75)	Cohort study (retrospective), Acute care hospitals	0.79
(47)	Case- control study (retrospective), Acute care hospitals	0.79
(54)	Cross- sectional study (Panel survey), Setting not reported	0.79
(71)	Case- control study (retrospective), Tertiary hospital	0.56
(59)	Case- control study, Tertiary hospital	0.89
(73)	Case- control study (retrospective), Tertiary hospital	0.79
(80)	Cross- sectional study (prospective), Tertiary hospital	0.79

(65)	Cohort study (retrospective), Setting not reported	0.79

[Note: Please refer to 'References' of main paper to get detail reference of the study reported in the study reference number above]
