# Supplementary material for: The economic burden of antibiotic resistance: A systematic review and meta-analysis
Source: PLoS One. 2023 May 8;18(5):e0285170. doi: 10.1371/journal.pone.0285170 (PMC10166566; doi:10.1371/journal.pone.0285170)
Supplement: S2 Table — (PDF) [file pone.0285170.s002.pdf]

Supplementary Table 2. Healthcare costs of resistant infections, susceptible infections and attributable costs

**First author & publication year**	**Study country**	**Cost for resistant infection (US$, adjusted for 2020)**	**Cost for susceptible infection (US$, adjusted for 2020)**	**Attributable costs (US$)**	**P value**
Huang et al. 2018	China	26276.0	14989.1	11286.9	p<0.05
Jiang et al. 2017	Taiwan, China	4362.7	1648.4	2714.3	p < 0.0001.
Klein et al. 2019	USA	42257.6	44629.0	-2371.4	p=0.045, (higher for MSSA)
Maslikowska et al. 2016	Canada	30986.6	8789.6	22197.0	p = 0.0391
Meng et al. 2017	China	12759.9	10362.9	2397.1	p= 0.05
Naylor et al. 2019	UK (England)	-	-	337.7	Not reported
Puchter et al. 2018	Germany	63448.2	34159.1	29289.1	p= 0.03
Thaden et la. 2017	USA	64362.9	39586.9	24776.0	P = 0.003
Judd et al. 2016	USA	49670.7	21179.4	28491.3	p<0.001
Stewardson et al. 2016	European countries	2016.9	1688.5	328.4	Reported not significant, but p value not reported
Tabak et al. 2019	USA	9494.9	7852.9	1642.0	p<0.001
Tabak et al. 2020	USA	38277.2	26521.2	11756.0	p=0.083
Thatrimontrichai et al. 2019	Thailand	9638.9	7347.5	2291.4	p= 0.01
Uematsu et al. 2018	Japan	15934.7	14307.1	1627.6	p<0.001
Jia et al. 2019	China	6838.5	2537.8	4300.7	p<0.01
Zhen et al. 2020	China	15453.3	4733.0	10720.3	p<0.001
Zilberberg et al. 2019	USA	82388.5	71498.3	10890.2	p<0.001
François et al. 2016	France	83.0	92.0	-9.0	p= 0.63
Uematsu et al. 2017	Japan	34051.2	10128.7	23922.5	Not reported
Giraldi et al. 2019	Italy	15810.5	-	16601.1	Not reported
Inagaki et al. 2019	USA	40732.1	40768.3	-36.2	P = 0.2
Tabak et al. 2019b	USA	88525.3	64231.5	24293.8	P = 0.002

Uematsu et al. 2016	Japan	13224.0	7472.0	5752.0	$p<0.001$
Thrope et al. 2017	USA	4052.5	1527.6	2524.9	$p<0.001$
Zhen et al. 2017	China	34121.9	22078.4	12043.5	$p<0.001$
Mora-Guzman et al., 2020	Spain	34663.8	20490.2	14173.6	$p= 0.064$
Zhen et al. 2021	China	14527.0	10481.4	4045.5	Not reported
Iskandar et al. 2021	Lebanon	8685.2	5851.0	2834.2	$p<0.001$
Nelson et al. 2021	USA	-	-	18034.1	Not reported
