# Supplementary material for: The economic burden of antibiotic resistance: A systematic review and meta-analysis
Source: PLoS One. 2023 May 8;18(5):e0285170. doi: 10.1371/journal.pone.0285170 (PMC10166566; doi:10.1371/journal.pone.0285170)
Supplement: S6 Table — (PDF) [file pone.0285170.s006.pdf]

Supplementary Table 6. Healthcare costs of resistant infections, susceptible infections and attributable costs by income category of countries

First author & publication year	Study country	Income category of the study country	Cost for resistant infection (US$, adjusted for 2020)	Cost for susceptible infection (US$, adjusted for 2020)	Attributable costs (US$)	P value
Jiang et al. 2017	Taiwan, China	HIE	4362.7	1648.4	2714.3	p < 0.0001.
Maslikowska et al. 2016	Canada	HIE	30986.6	8789.6	22197.0	p = 0.0391
Puchter et al. 2018	Germany	HIE	63448.2	34159.1	29289.1	p= 0.03
Klein et al. 2019	USA	HIE	42257.6	44629.0	-2371.4	p=0.045, (higher for MSSA)
Thaden et la. 2017	USA	HIE	64362.9	39586.9	24776.0	P = 0.003
Judd et al. 2016	USA	HIE	49670.7	21179.4	28491.3	p<0.001
Stewardson et al. 2016	European countries	HIE	2016.9	1688.5	328.4	Reported not significant, but p value not reported
Tabak et al. 2019	USA	HIE	9494.9	7852.9	1642.0	p<0.001

First author & publication year	Study country	Income category of the study country	Cost for resistant infection (US$, adjusted for 2020)	Cost for susceptible infection (US$, adjusted for 2020)	Attributable costs (US$)	P value
Tabak et al. 2020	USA	HIE	38277.2	26521.2	11756.0	p=0.083
Uematsu et al. 2018	Japan	HIE	15934.7	14307.1	1627.6	p<0.001
Zilberberg et al. 2019	USA	HIE	82388.5	71498.3	10890.2	p<0.001
Uematsu et al. 2017	Japan	HIE	34051.2	10128.7	23922.5	Not reported
Giraldi et al. 2019	Italy	HIE	15810.5	-	16601.1	Not reported
Inagaki et al. 2019	USA	HIE	40732.1	40768.3	-36.2	P = 0.2
Tabak et al. 2019b	USA	HIE	88525.3	64231.5	24293.8	P = 0.002
Uematsu et al. 2016	Japan	HIE	13224.0	7472.0	5752.0	p<0.001
Mora-Guzman et al., 2020	Spain	HIE	34663.8	20490.2	14173.6	p= 0.064
Nelson et al. 2021	USA	HIE	-	-	18034.12	Not reported
Naylor et al. 2019	UK (England)	HIE	-	-	337.7	Not reported
François et al. 2016	France	HIE	83.0	92.0	-9.0	p= 0.63
Thrope et al. 2017	USA	HIE	4052.5	1527.6	2524.9	p<0.001
Meng et al. 2017	China	UMIE	12759.9	10362.9	2397.1	p= 0.05
