# Supplementary material for: The economic burden of antibiotic resistance: A systematic review and meta-analysis
Source: PLoS One. 2023 May 8;18(5):e0285170. doi: 10.1371/journal.pone.0285170 (PMC10166566; doi:10.1371/journal.pone.0285170)
Supplement: S7 Table — (PDF) [file pone.0285170.s007.pdf]

Supplementary Table 7. Segregated costs for resistant and susceptible infection

**Author, publication year & study country**	**Cost items**	**Adjusted costs for resistant infection ($) (sample size)**	**Adjusted costs for susceptible infection ($) (sample size)**	**Adjusted attributable cost ($)**
Huang et al. 2018, China		Medical Costs of Patients with CRKP (n=267)	Medical Costs of Patients with CSKP (n=1328)	
	Medicine	9972.2	4147.3	5824.9
	Antibacterial	2335.5	1018.9	1316.6
	Therapies	8186.8	3995.2	4191.6
	Lab & radiology	3325.8	2228.8	1097.0
	Nursing & monitoring	1147.7	527.9	619.8
	Bed & board	429.7	300.9	128.8
Meng et al. 2017, China		Medical costs of CREC group (n=49)	Medical costs of CSEC group (n=98)	
	Total costs	12805.7	10400.0	2405.6
	Examination costs	474.4	417.3	57.1
	Medical test costs	1027.2	754.5	272.7
	Total drug costs	6911.8	4715.0	2196.8
	Anti-infective drug costs	1445.6	782.3	663.3
Jia et al. 2019, China		Medical cost of MDRO (n=331)	Medical cost of non-MDRO (n=331)	
	Medication cost	2465.2	838.4	1626.8
	Antimicrobial cost	620.8	210.7	410.1
	Therapeutic cost	536.9	159.4	377.4
	Laboratory cost	529.6	234.1	295.5
	Bed cost	184.8	65.6	119.2
	Operating cost	458.3	359.1	99.3
	Inspection cost	229.7	130.8	98.8
	Nursing cost	120.7	37.8	82.9

**Authors, publication year, & study country**	**Cost items**	**Adjusted medical costs for resistant infection ($) (sample size)**	**Adjusted medical costs for susceptible infection ($) (sample size)**	**Adjusted attributable cost ($)**
Zhen et al. 2020, China		Treatment costs for MRSA (n=1335)	Treatment costs for MSSA (n=1397)	
	Total hospital cost	15453.3	4733.0	10720.3
	Antibiotic cost	1491.0	251.1	1239.9
	Medication cost	7963.8	1806.8	6157.0
	Diagnostic cost	2082.5	1102.6	979.8
	Treatment cost	3047.8	999.9	2047.9
	Material cost	1013.3	264.5	748.8
	Other cost	23.4	15.6	7.8
Thorpe et al. 2017, USA		Bacterial infections without antibiotic resistance (n=123254)	Bacterial infections with antibiotic resistance (n=12766374)	
	All cost	4052.5	1527.6	2524.9
	Inpatient	2558.8	907.4	1651.5
	Office-based	561.1	212.6	348.5
	Emergency department	274.0	144.7	129.3
	Prescription drugs	334.2	105.2	229.0
	Outpatient	124.9	84.4	40.5
	Home health	198.4	74.5	123.8
Zhen et al. 2017, China		Medical costs for CRAB infection (n=2126)	Medical costs for CRAB infection (n=854)	
	Total medical cost	34121.9	22078.4	12043.5
	medication cost	13070.4	8064.4	5006.0
	antibiotics cost	3400.8	1888.1	1512.7
	laboratory cost	4057.8	2677.5	1380.4
	hospital cost	11566.7	6835.5	4731.2

Authors, publication year, & study country	Cost items	Adjusted medical costs for resistant infection ($) (sample size)	Adjusted medical costs for susceptible infection ($) (sample size)	Adjusted attributable cost ($)
Iskandar et al. 2021, Lebanon		Hospitalisation costs of resistant bacterial infections (n=911)	Hospitalisation costs of susceptible bacterial infections (n=854)	
	Pharmaceuticals charges	3757.9	2091.1	1666.8
	Antibiotics charges	1236.4	546.1	690.3
	Medical accessories charges-	469.0	335.0	134.0
	Oxygen charges-	268.0	109.6	158.4
	Imaging charges	645.6	562.4	83.2
	Laboratory and microbiology charges	1762.2	1449.6	312.7
	Third-party payment	7593.0	5211.5	2381.4
	Patient co-payment	1041.5	692.3	349.2
	Total charges from index date	8685.2	5851.0	2834.2
