# Supplementary material for: The economic burden of antibiotic resistance: A systematic review and meta-analysis
Source: PLoS One. 2023 May 8;18(5):e0285170. doi: 10.1371/journal.pone.0285170 (PMC10166566; doi:10.1371/journal.pone.0285170)
Supplement: S8 Table — (PDF) [file pone.0285170.s008.pdf]

Supplementary Table 8. Length of stay for resistant and susceptible infections (weighted length of stay and p-values)

First author & publication year	Study design	Healthcare settings	Study country	Income category of study country	Perspective of study	Mean LoS resistant infections	Mean weighted LoS (Resistant infections)	Mean LoS susceptible infections	Mean weighted LoS (Susceptible infections)	Excess LoS (weighted)	P - value
Huang et al. 2018	Case - control study	Tertiary hospital	China	UMIE	Healthcare/ hospital	31.0	31.0	24	24.0	7.00	p<0.001
Jiang et al. 2017	Case - control study	Not reported	China	UMIE	Healthcare/ hospital	32.5	30.2	24	22.5	7.73	p=0.082
Klein et al. 2019	Cohort study	Secondary + tertiary hospitals	USA	HIE	Healthcare/ hospital	7.1	7.7	8	8.4	-0.71	Not reported
Maslikowska et al. 2016	Case - control study	Tertiary hospital	Canada	HIE	Healthcare/ hospital	12.6	11.9	11	10.7	1.2	p=0.0236
Naylor et al. 2019	Cohort study	Secondary healthcare setting	UK	HIE	Healthcare/ hospital	10.0	10.9	7	7.6	3.3	significant, but value not reported
Puchter et al. 2018	Case - control study	Tertiary hospital	Germany	HIE	Healthcare/ hospital	36.5	30.8	25	21.3	9.5	p=0.112
Thaden et la. 2017	Cohort study	Tertiary hospital	USA	HIE	Healthcare/ hospital	13.0	13.8	10	10.3	3.4	p= 0.0005
Judd et al. 2016	Cohort study	Tertiary hospital	USA	HIE	Healthcare/ hospital	17.2	15.4	10	8.7	6.7	p= 0.004
Tabak et al. 2019	Cohort study	Acute care hospitals	USA	HIE	Healthcare/ hospital	7.2	7.6	6	6.4	1.3	p<0.001
Tabak et al. 2020	Cohort study	Acute care hospitals	USA	HIE	Healthcare/ hospital	18.5	15.6	16	13.2	2.5	p=0.249
Thatrimontrichai et al. 2019	Case - control study	Tertiary hospital	Thailand	UMIE	Healthcare/ hospital	49.4	48.6	48	47.0	1.6	p<0.001
Uematsu et al. 2018	Cohort study	Acute care hospitals	Japan	HIE	Healthcare/ hospital	32.0	31.5	29	28.2	3.2	p<0.001

First author & publication year	Study design	Healthcare settings	Study country	Income category of study country	Perspective of study	Mean LoS resistant infections	Mean weighted LoS (Resistant infections)	Mean LoS susceptible infections	Mean weighted LoS (Susceptible infections)	Excess LoS (weighted)	P - value
Zhen et al. 2020	Case - control study	Tertiary hospital	China	UMIE	Healthcare/ hospital	27.0	27.0	21	21.0	6.0	p<0.001
Zilberberg et al. 2019	Cohort study	Not reported	USA	HIE	Healthcare/ hospital	25.8	27.9	22	23.9	4.0	p<0.001
Uematsu et al. 2017	Case - control study	Acute care hospitals	Japan	HIE	Payer's perspective	56.6	58.1	18	18.9	39.2	p value not reported
Giraldi et al. 2019	Cohort study	Tertiary hospital	Italy	HIE	Healthcare/ hospital	43.6	43.0	25	24.4	18.5	p<0.001
Tabak et al. 2019b	Cohort study	Acute care hospitals	USA	HIE	Healthcare/ hospital	29.1	31.2	22	24.0	7.2	p<0.001
Uematsu et al. 2016	Case - control study	Acute care hospitals	Japan	HIE	Healthcare/ hospital	30.1	32.5	21	22.7	9.8	p<0.001
Mora-Guzman et al., 2020	Case - control study	Tertiary hospital	Spain	HIE	Healthcare/ hospital	44.8	39.9	27	23.8	16.2	p<0.001
Zhen et al. 2021	Case - control study	Tertiary hospital	China	UMIE	Healthcare/ hospital	27.7	30.1	22	24.1	6.0	Not reported
Iskandar et al. 2021	Cross - sectional study	Tertiary hospital	Lebanon	LMIE	Payer's perspective	8.4	8.8	6	6.7	2.2	P<0.001

[Note: lower-middle income economy (LMIE), upper-middle income economy (UMIE), high-income economy (HIE)]
