# Supplementary material for: The economic burden of antibiotic resistance: A systematic review and meta-analysis
Source: PLoS One. 2023 May 8;18(5):e0285170. doi: 10.1371/journal.pone.0285170 (PMC10166566; doi:10.1371/journal.pone.0285170)
Supplement: S9 Table — (PDF) [file pone.0285170.s009.pdf]

Supplementary Table 9. Mortality rate and odds ratios for resistant and susceptible infections (weighted and p-values)

First author & publication year	Study Design	Study Setting	Study country	Income Category	Study Perspective	Mortality%-Resistant infections	Weighted Mortality-Resistant infections	Mortality %-Susceptible infections	Weighted Mortality-Susceptible infections	Weighted excess mortality %	P- value
Huang et al. 2018	Case - control study	Tertiary hospital	China	UMIE	Healthcare/ Hospital	13.5	15.2	10.6	11.9	3.3	p<0.324
Klein et al. 2019	Cohort study	Tertiary + secondary hospital	USA	HIE	Healthcare/ Hospital	4.6	4.3	4.4	4.2	0.2	p<0.001
Maslikowska et al. 2016	Case - control study	Tertiary hospital	Canada	HIE	Healthcare/ Hospital	17.3	15.1	5.3	4.6	10.5	p= 0.0368
Meng et al. 2017	Case - control study	Tertiary hospital	China	UMIE	Healthcare/ Hospital	12.0	13.2	1.0	1.1	12.1	p=0.01
Puchter et al. 2018	Case - control study	Tertiary hospital	German	HIE	Healthcare/ Hospital	33.3	39.1	26.2	30.8	8.3	p= 0.634
Stewardson et al. 2016	Cohort study	Tertiary + secondary hospital	Europe	HIE	Healthcare/ Hospital	22.1	25.2	16.8	19.2	6.0	not significant but not reported
Uematsu et al. 2018	Cohort study	Not reported or Acute care	Japan	HIE	Healthcare/ Hospital	17.0	15.8	13.0	12.1	3.7	p<0001
Zhen et al. 2020	Case - control study	Tertiary hospital	China	UMIE	Healthcare/ Hospital	3.0	3.4	4.0	4.5	-1.1	p=0.265
Zilberberg et al. 2019	Cohort study	Not reported or Acute care	USA	HIE	Healthcare/ Hospital	22.9	25.7	21.6	24.2	1.5	p= 0.223
Uematsu et al. 2017	Case - control study	Not reported or Acute care	Japan	HIE	Payer's perspective	22.9	26.9	6.3	7.4	19.6	Not reported
Giraldi et al. 2019	Cohort study	Tertiary hospital	Italy	HIE	Healthcare/ Hospital	32.8	27.6	10.7	9.0	18.6	p= 0.0001
Uematsu et al. 2016	Case - control study	Not reported or Acute care	Japan	HIE	Healthcare/ Hospital	22.6	11.4	12.2	6.2	5.2	p<0.001
Zhen et al. 2021	Case - control study	Tertiary hospital	China	UMIE	Healthcare/ Hospital	3.6	3.4	2.1	1.9	1.4	Not reported

						Odds ratio	Lower bound at 95%	Upper bound at 95%			P- value
Jiang et al. 2017	Case - control study	Not reported or Acute care	China	UMIE	Healthcare/ Hospital	2.7	1.04	7.14			p=0.003
Thaden et al. 2017	Cohort study	Tertiary hospital	USA	HIE	Healthcare/ Hospital	1.28	0.87	1.89			P= 0.12
Judd et al. 2016	Cohort study	Tertiary hospital	USA	HIE	Healthcare/ Hospital	2.89	1.15	7.28			p=0.003
Tabak et al. 2019	Cohort study	Not reported or Acute care	USA	HIE	Healthcare/ Hospital	1.58	1.14	2.2			p<0.01
Tabak et al. 2020	Cohort study	Not reported or Acute care	USA	HIE	Healthcare/ Hospital	1.23	0.41	3.69			p=0.708
Thatrimontrichai et al. 2019	Case - control study	Tertiary hospital	Thailand	UMIE	Healthcare/ Hospital	3.63	2.21	5.98			p<0.001
Inagaki et al. 2019	Cohort study	Tertiary + secondary hospital	USA	HIE	Healthcare/ Hospital	1.15	1.07	1.23			p<0.01

[Note: upper-middle income economy (UMIE), high-income economy (HIE)]
