# Supplementary material for: The economic burden of antibiotic resistance: A systematic review and meta-analysis
Source: PLoS One. 2023 May 8;18(5):e0285170. doi: 10.1371/journal.pone.0285170 (PMC10166566; doi:10.1371/journal.pone.0285170)
Supplement: S10 Table — (PDF) [file pone.0285170.s010.pdf]

Supplementary Table 10. Comparisons of mean mortality % between resistant and susceptible infections

SN	Description of the variables	Mean mortality (%)*	95% confidence	
			Lower bound (%)	Upper bound (%)
**1.**	**Overall mortality rate**			
	Resistant infection (n = 13)	17.4	10.8	24.0
	Susceptible infection (n = 13)	10.5	5.1	16.0
	Excess mortality (n = 13)	6.9	2.8	10.9

** Weighted mean mortality % were calculated using random weight (relative weight) of each study.*
