# Supplementary material for: The economic burden of antibiotic resistance: A systematic review and meta-analysis
Source: PLoS One. 2023 May 8;18(5):e0285170. doi: 10.1371/journal.pone.0285170 (PMC10166566; doi:10.1371/journal.pone.0285170)
Supplement: S11 Table — (PDF) [file pone.0285170.s011.pdf]

Supplementary Table 11. Readmission rate and odds ratios for resistant and susceptible infections (weighted and p-values)

**Name of first author & publication year**	**Study Design**	**Study Setting**	**Study country**	**Income Category**	**Study Perspective**	**Exposure group readmission %**	**Control group readmission%**	**Weighted readmission %- resistant infections**	**Weighted readmission %- susceptible infections**	**Excess readmission %**	**P- value**
Zilberberg et al. 2019	Cohort study	Not reported	USA	HIE	Healthcare/ hospital	23.5	22.0	16.4	15.4	1.0	p= 0.748
Tabak et al. 2019b	Cohort study	Acute care hospitals	USA	HIE	Healthcare/ hospital	16.2	11.1	33.5	22.9	10.5	p=0.006
Mora-Guzman et al., 2020	Case – control	Tertiary hospital	Spain	HIE	Healthcare/ hospital	27.5	16.7	6.6	4.0	2.6	p = 0.223
						**Odds ratio**	**Lower CI at 95%**	**Upper CI at 95%**			**P- value**
Tabak et al. 2019	Cohort study	Acute care hospitals	USA	HIE	Healthcare/ hospital	1.55	1.11	2.16			p<0.05
Tabak et al. 2020	Cohort study	Acute care hospitals	USA	HIE	Healthcare/ hospital	1.52	0.34	6.72			p=0.58

[Note: high income economy (HIE)]
