# Supplementary material for: The economic burden of antibiotic resistance: A systematic review and meta-analysis
Source: PLoS One. 2023 May 8;18(5):e0285170. doi: 10.1371/journal.pone.0285170 (PMC10166566; doi:10.1371/journal.pone.0285170)
Supplement: S12 Table — (PDF) [file pone.0285170.s012.pdf]

Supplementary Table 12. Comparisons of readmission % between resistant and susceptible infections

SN	Description of the variables	Mean readmission (%)*	95% confidence	
			Lower bound (%) **	Upper bound (%) **
**1.**	**Overall readmission rate**			
	Resistant infection (n = 3)	18.8	-	-
	Susceptible infection (n = 3)	14.1	-	-
	Excess readmission to resistant infection (n = 3)	4.7	-	-

** Weighted mean readmission % were calculated using random weight (relative weight) of each study.*

***Did not calculate 95% confidence interval (lower and upper bounds) in these groups as there were only limited number of studies (three) available.*
