# Supplementary material for: The economic burden of antibiotic resistance: A systematic review and meta-analysis
Source: PLoS One. 2023 May 8;18(5):e0285170. doi: 10.1371/journal.pone.0285170 (PMC10166566; doi:10.1371/journal.pone.0285170)
Supplement: S1 Fig — (PDF) [file pone.0285170.s013.pdf]

Supplementary Figure 1. Impact of resistant infections on length of stay at hospital

Impact of resistant infections on length of stay

Study name	Hedges's g	Lower limit	Upper limit	p-Value
Jiang et al. 2017	0.293	-0.034	0.620	0.080
Klein et al. 2019	-0.118	-0.123	-0.113	0.000
Maslikowska et al. 2016	0.069	-0.250	0.388	0.671
Naylor et al. 2019	0.334	0.290	0.378	0.000
Puchter et al. 2018	0.381	-0.047	0.809	0.081
Thaden et la. 2017	0.305	0.172	0.438	0.000
Judd et al. 2016	0.879	0.512	1.246	0.000
Tabak et al. 2019	0.231	0.101	0.361	0.000
Tabak et al. 2020	0.176	-0.248	0.601	0.416
Thatrimontrichai et al. 2019	0.043	-0.217	0.304	0.744
Uematsu et al. 2018	0.152	-0.109	0.413	0.253
Zilberberg et al. 2019	0.285	0.221	0.349	0.000
Uematsu et al. 2017	1.113	0.915	1.311	0.000
Giraldi et al. 2019	0.696	0.438	0.953	0.000
Tabak et al. 2019b	0.185	0.084	0.285	0.000
Uematsu et al. 2016	0.395	0.317	0.473	0.000
Mora-Guzman et al., 2020	1.004	0.632	1.377	0.000
Zhen et al. 2021	0.716	0.664	0.768	0.000
Iskandar et al. 2021	0.311	0.156	0.467	0.000
	0.387	0.198	0.576	0.000

Hedges's g and 95% CI

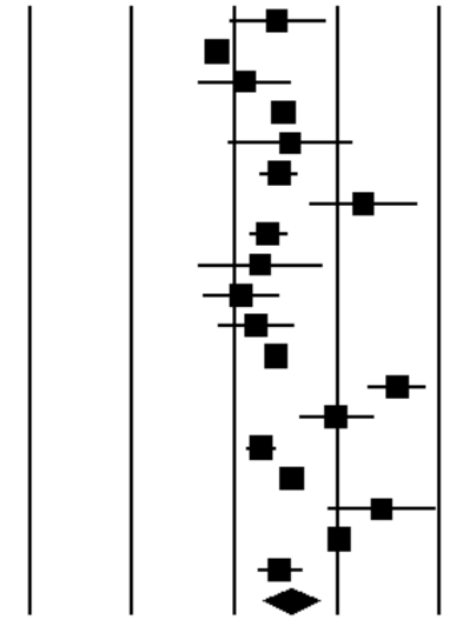

Meta Analysis- Random Effects Model

Model	Number Studies	Point estimate	Standard error	Variance	Lower limit	Upper limit	Z-value	P-value	Q-value	df (Q)	P-value	I-squared	Tau Squared	Standard Error	Variance	Tau
Model	Effect size and 95% confidence interval						Test of null (2-Tail)		Heterogeneity				Tau-squared			
Fixed	19	-0.096	0.003	0.000	-0.101	-0.091	-37.896	0.000	2043.832	18	0.000	99.119	0.162	0.141	0.020	0.403
Random	19	0.387	0.096	0.009	0.198	0.576	4.012	0.000								
