# Supplementary material for: The economic burden of antibiotic resistance: A systematic review and meta-analysis
Source: PLoS One. 2023 May 8;18(5):e0285170. doi: 10.1371/journal.pone.0285170 (PMC10166566; doi:10.1371/journal.pone.0285170)
Supplement: S2 Fig — (PDF) [file pone.0285170.s014.pdf]

Supplementary Figure 2. Impact of resistant infections on length of stay at hospital by study perspective

Impact of resistant infections on length of stay by study perspective

Study name	Group by Study perspective	Hedges's g	Lower limit	Upper limit	p-Value
Jiang et al. 2017	Healthcare/hospital	0.293	-0.034	0.620	0.080
Klein et al. 2019	Healthcare/hospital	-0.118	-0.123	-0.113	0.000
Maslikowska et al. 2016	Healthcare/hospital	0.069	-0.250	0.388	0.671
Naylor et al. 2019	Healthcare/hospital	0.334	0.290	0.378	0.000
Puchter et al. 2018	Healthcare/hospital	0.381	-0.047	0.809	0.081
Thaden et la. 2017	Healthcare/hospital	0.305	0.172	0.438	0.000
Judd et al. 2016	Healthcare/hospital	0.879	0.512	1.246	0.000
Tabak et al. 2019	Healthcare/hospital	0.231	0.101	0.361	0.000
Tabak et al. 2020	Healthcare/hospital	0.176	-0.248	0.601	0.416
Thatrimontrichai et al. 2019	Healthcare/hospital	0.043	-0.217	0.304	0.744
Uematsu et al. 2018	Healthcare/hospital	0.152	-0.109	0.413	0.253
Zilberberg et al. 2019	Healthcare/hospital	0.285	0.221	0.349	0.000
Giraldi et al. 2019	Healthcare/hospital	0.696	0.438	0.953	0.000
Tabak et al. 2019b	Healthcare/hospital	0.185	0.084	0.285	0.000
Uematsu et al. 2016	Healthcare/hospital	0.395	0.317	0.473	0.000
Mora-Guzman et al., 2020	Healthcare/hospital	1.004	0.632	1.377	0.000
Zhen et al. 2021	Healthcare/hospital	0.716	0.664	0.768	0.000
	Healthcare/hospital	0.347	0.151	0.544	0.001
Uematsu et al. 2017	Payer's perspective	1.113	0.915	1.311	0.000
Iskandar et al. 2021	Payer's perspective	0.311	0.156	0.467	0.000
	Payer's perspective	0.710	-0.076	1.495	0.077

Hedges's g and 95% CI

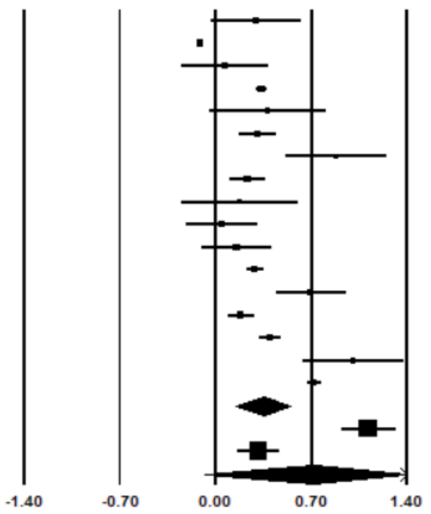

Meta Analysis- Random Effects Model

Groups		Effect size and 95% confidence interval					Test of null (2-Tail)		Heterogeneity				Tau-squared			
Group	Number Studies	Point estimate	Standard error	Variance	Lower limit	Upper limit	Z-value	P-value	Q-value	df (Q)	P-value	I-squared	Tau Squared	Standard Error	Variance	Tau
**Fixed effect analysis**																
Healthcare/hos	17	-0.097	0.003	0.000	-0.102	-0.092	-38.330	0.000	1873.670	16	0.000	99.146	0.155	0.137	0.019	0.393
Payer's	2	0.617	0.062	0.004	0.495	0.739	9.901	0.000	39.054	1	0.000	97.439	0.313	0.454	0.206	0.559
Total within									1912.725	17	0.000					
Total between									131.107	1	0.000					
**Random effects analysis**																
Healthcare/hos	17	0.347	0.100	0.010	0.151	0.544	3.472	0.001								
Payer's	2	0.710	0.401	0.161	-0.076	1.495	1.771	0.077								
Total between									0.769	1	0.380					
