# Supplementary material for: The economic burden of antibiotic resistance: A systematic review and meta-analysis
Source: PLoS One. 2023 May 8;18(5):e0285170. doi: 10.1371/journal.pone.0285170 (PMC10166566; doi:10.1371/journal.pone.0285170)
Supplement: S3 Fig — (PDF) [file pone.0285170.s015.pdf]

Supplementary file 3. Impact of resistant infections on length of stay at hospital by healthcare setting

Impact of resistant infections on length of stay by healthcare settings

Study name	Group by Healthcare setting	Hedges's g	Lower limit	Upper limit	p-Value
Klein et al. 2019	Secondary + tertiary hospitals	-0.118	-0.123	-0.113	0.000
	Secondary + tertiary hospitals	-0.118	-0.123	-0.113	0.000
Naylor et al. 2019	Secondary healthcare setting	0.334	0.290	0.378	0.000
	Secondary healthcare setting	0.334	0.290	0.378	0.000
Maslikowska et al. 2016	Tertiary hospital	0.069	-0.250	0.388	0.671
Puchter et al. 2018	Tertiary hospital	0.381	-0.047	0.809	0.081
Thaden et la. 2017	Tertiary hospital	0.305	0.172	0.438	0.000
Judd et al. 2016	Tertiary hospital	0.879	0.512	1.246	0.000
Thatrimontrichai et al. 2019	Tertiary hospital	0.043	-0.217	0.304	0.744
Giraldi et al. 2019	Tertiary hospital	0.696	0.438	0.953	0.000
Mora-Guzman et al., 2020	Tertiary hospital	1.004	0.632	1.377	0.000
Iskandar et al. 2021	Tertiary hospital	0.311	0.156	0.467	0.000
Zhen et al. 2021	Tertiary hospital	0.716	0.664	0.768	0.000
	Tertiary hospital	0.480	0.274	0.686	0.000

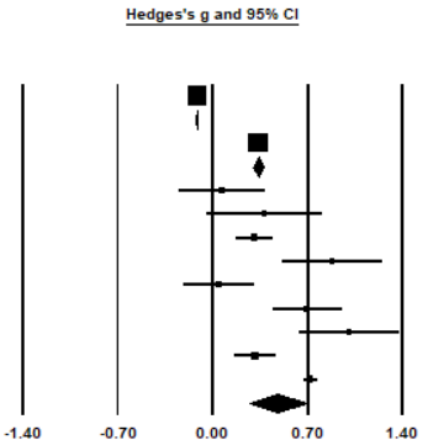

Meta Analysis- Random Effects Model

Groups		Effect size and 95% confidence interval					Test of null (2-Tail)		Heterogeneity				Tau-squared			
Group	Number Studies	Point estimate	Standard error	Variance	Lower limit	Upper limit	Z-value	P-value	Q-value	df (Q)	P-value	I-squared	Tau Squared	Standard Error	Variance	Tau
**Fixed effect analysis**																
Secondary +	1	-0.118	0.003	0.000	-0.123	-0.113	-45.625	0.000	0.000	0	1.000	0.000	0.000	0.000	0.000	0.000
Secondary	1	0.334	0.022	0.001	0.290	0.378	14.900	0.000	0.000	0	1.000	0.000	0.000	0.000	0.000	0.000
Tertiary hospital	9	0.612	0.022	0.000	0.568	0.655	27.627	0.000	87.757	8	0.000	90.884	0.080	0.069	0.005	0.283
Total within									87.757	8	0.000					
Total between									1455.253	2	0.000					
**Random effects analysis**																
Secondary +	1	-0.118	0.003	0.000	-0.123	-0.113	-45.625	0.000								
Secondary	1	0.334	0.022	0.001	0.290	0.378	14.900	0.000								
Tertiary hospital	9	0.480	0.105	0.011	0.274	0.686	4.568	0.000								
Total between									432.782	2	0.000					
