# Supplementary material for: The economic burden of antibiotic resistance: A systematic review and meta-analysis
Source: PLoS One. 2023 May 8;18(5):e0285170. doi: 10.1371/journal.pone.0285170 (PMC10166566; doi:10.1371/journal.pone.0285170)
Supplement: S4 Fig — (PDF) [file pone.0285170.s016.pdf]

Supplementary file 4. Impact of resistant infections on length of stay at hospital by income category of country

Impact of resistant infections on length of stay by income category of country

Study name	Group by World Bank-Income category	Hedges's g	Lower limit	Upper limit	p-Value
Klein et al. 2019	HIE	-0.118	-0.123	-0.113	0.000
Maslikowska et al. 2016	HIE	0.069	-0.250	0.388	0.671
Naylor et al. 2019	HIE	0.334	0.290	0.378	0.000
Puchter et al. 2018	HIE	0.381	-0.047	0.809	0.081
Thaden et la. 2017	HIE	0.305	0.172	0.438	0.000
Judd et al. 2016	HIE	0.879	0.512	1.246	0.000
Tabak et al. 2019	HIE	0.231	0.101	0.361	0.000
Tabak et al. 2020	HIE	0.176	-0.248	0.601	0.416
Uematsu et al. 2018	HIE	0.152	-0.109	0.413	0.253
Zilberberg et al. 2019	HIE	0.285	0.221	0.349	0.000
Uematsu et al. 2017	HIE	1.113	0.915	1.311	0.000
Giraldi et al. 2019	HIE	0.696	0.438	0.953	0.000
Tabak et al. 2019b	HIE	0.185	0.084	0.285	0.000
Uematsu et al. 2016	HIE	0.395	0.317	0.473	0.000
Mora-Guzman et al., 2020	HIE	1.004	0.632	1.377	0.000
	HIE	0.393	0.213	0.574	0.000
Jiang et al. 2017	UMIE	0.293	-0.034	0.620	0.080
Thatrimontrichai et al. 2019	UMIE	0.043	-0.217	0.304	0.744
Zhen et al. 2021	UMIE	0.716	0.664	0.768	0.000
Iskandar et al. 2021	UMIE	0.311	0.156	0.467	0.000
	UMIE	0.355	0.021	0.690	0.037

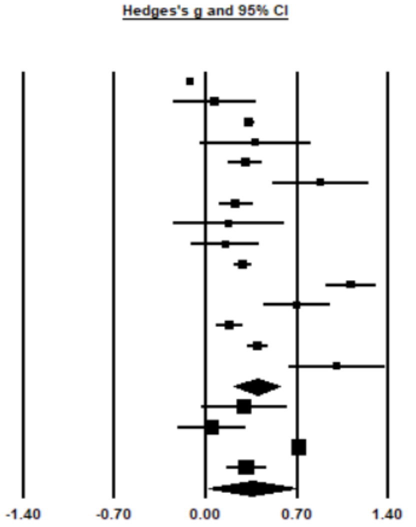

Meta Analysis- Random Effects Model

Groups		Effect size and 95% confidence interval					Test of null (2-Tail)		Heterogeneity				Tau-squared			
Group	Number Studies	Point estimate	Standard error	Variance	Lower limit	Upper limit	Z-value	P-value	Q-value	df (Q)	P-value	I-squared	Tau Squared	Standard Error	Variance	Tau
**Fixed effect analysis**																
HIE	15	-0.104	0.003	0.000	-0.109	-0.099	-40.871	0.000	1056.902	14	0.000	98.675	0.113	0.104	0.011	0.336
UMIE	4	0.646	0.024	0.001	0.598	0.694	26.508	0.000	49.915	3	0.000	93.990	0.105	0.116	0.013	0.323
Total within									1106.817	17	0.000					
Total between									937.015	1	0.000					
**Random effects analysis**																
HIE	15	0.393	0.092	0.008	0.213	0.574	4.269	0.000								
UMIE	4	0.355	0.171	0.029	0.021	0.690	2.080	0.037								
Total between									0.038	1	0.845					
