# Supplementary material for: The economic burden of antibiotic resistance: A systematic review and meta-analysis
Source: PLoS One. 2023 May 8;18(5):e0285170. doi: 10.1371/journal.pone.0285170 (PMC10166566; doi:10.1371/journal.pone.0285170)
Supplement: S5 Fig — (PDF) [file pone.0285170.s017.pdf]

Supplementary Figure 5. Impact of resistant infections on mortality

Impact of resistant infections on mortality

Study name	Statistics for each study			
	Odds ratio	Lower limit	Upper limit	p-Value
Thaden et al. 2017	1.280	0.868	1.887	0.212
Judd et al. 2016	2.890	1.149	7.271	0.024
Tabak et al. 2020	1.230	0.410	3.690	0.712
Thatrimontrichai et al. 2019	3.630	2.207	5.971	0.000
Inagaki et al. 2019	1.150	1.073	1.233	0.000
Tabak et al. 2019	1.580	1.137	2.195	0.006
Jiang et al. 2017	2.700	1.030	7.075	0.043
Huang et al. 2018	1.322	0.893	1.958	0.163
Klein et al. 2019	1.048	1.022	1.074	0.000
Maslikowska et al. 2016	3.722	1.154	12.007	0.028
Meng et al. 2017	13.256	1.548	113.508	0.018
Puchter et al. 2018	1.409	0.550	3.609	0.475
Stewardson et al. 2016	1.400	0.929	2.109	0.107
Uematsu et al. 2018	1.371	1.252	1.501	0.000
Zhen et al. 2020	0.759	0.504	1.143	0.187
Uematsu et al. 2017	4.448	4.376	4.520	0.000
Giraldi et al. 2019	4.090	2.055	8.140	0.000
Uematsu et al. 2016	2.096	1.738	2.528	0.000
Zhen et al. 2021	1.757	1.283	2.406	0.000
Zilberberg et al. 2019	1.081	0.927	1.259	0.321
	1.844	1.187	2.865	0.006

Odds ratio and 95% CI

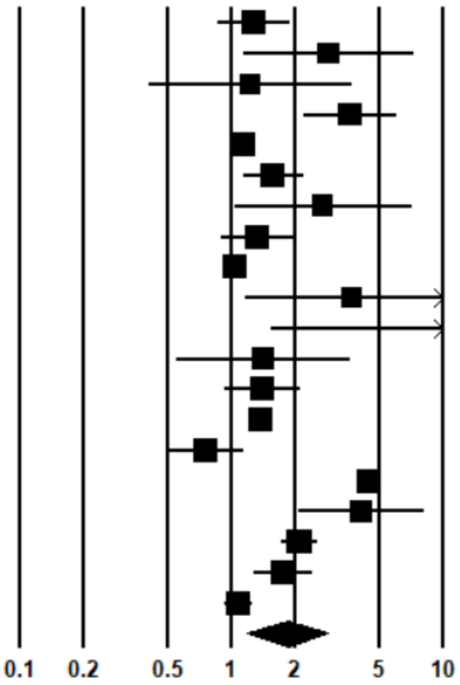

Meta Analysis- Random Effects Model

Model		Effect size and 95% interval			Test of null (2-Tail)		Heterogeneity				Tau-squared			
Model	Number Studies	Point estimate	Lower limit	Upper limit	Z-value	P-value	Q-value	df (Q)	P-value	I-squared	Tau Squared	Standard Error	Variance	Tau
Fixed	20	2.696	2.661	2.731	149.850	0.000	10444.317	19	0.000	99.818	0.901	1.013	1.026	0.949
Random	20	1.844	1.187	2.865	2.722	0.006								
