# Supplementary material for: The economic burden of antibiotic resistance: A systematic review and meta-analysis
Source: PLoS One. 2023 May 8;18(5):e0285170. doi: 10.1371/journal.pone.0285170 (PMC10166566; doi:10.1371/journal.pone.0285170)
Supplement: S6 Fig — (PDF) [file pone.0285170.s018.pdf]

Supplementary Figure 6. Impact of resistant infections on mortality by study perspective

Impact of resistant infections on mortality- by study perspective

Study name	Group by Study perspective	Odds ratio	Lower limit	Upper limit	p-Value
Thaden et al. 2017	Healthcare/hospital	1.280	0.868	1.887	0.212
Judd et al. 2016	Healthcare/hospital	2.890	1.149	7.271	0.024
Tabak et al. 2020	Healthcare/hospital	1.230	0.410	3.690	0.712
Thatrimontrichai et al. 2019	Healthcare/hospital	3.630	2.207	5.971	0.000
Inagaki et al. 2019	Healthcare/hospital	1.150	1.073	1.233	0.000
Tabak et al. 2019	Healthcare/hospital	1.580	1.137	2.195	0.006
Jiang et al. 2017	Healthcare/hospital	2.700	1.030	7.075	0.043
Huang et al. 2018	Healthcare/hospital	1.322	0.893	1.958	0.163
Klein et al. 2019	Healthcare/hospital	1.048	1.022	1.074	0.000
Maslikowska et al. 2016	Healthcare/hospital	3.722	1.154	12.007	0.028
Meng et al. 2017	Healthcare/hospital	13.256	1.548	113.508	0.018
Puchter et al. 2018	Healthcare/hospital	1.409	0.550	3.609	0.475
Stewardson et al. 2016	Healthcare/hospital	1.400	0.929	2.109	0.107
Uematsu et al. 2018	Healthcare/hospital	1.371	1.252	1.501	0.000
Zhen et al. 2020	Healthcare/hospital	0.759	0.504	1.143	0.187
Giraldi et al. 2019	Healthcare/hospital	4.090	2.055	8.140	0.000
Uematsu et al. 2016	Healthcare/hospital	2.096	1.738	2.528	0.000
Zhen et al. 2021	Healthcare/hospital	1.757	1.283	2.406	0.000
Zilberberg et al. 2019	Healthcare/hospital	1.081	0.927	1.259	0.321
	Healthcare/hospital	1.481	1.292	1.699	0.000
Uematsu et al. 2017	Payer's perspective	4.448	4.376	4.520	0.000
	Payer's perspective	4.448	4.376	4.520	0.000

Odds ratio and 95% CI

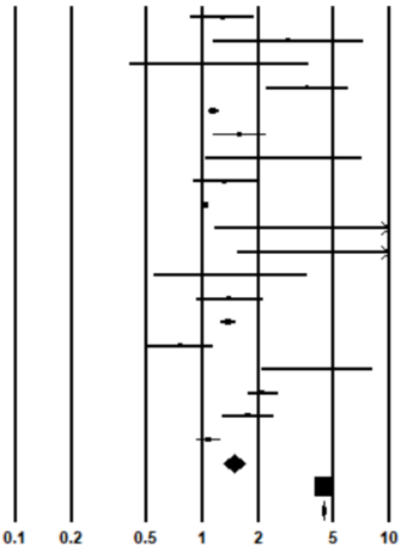

Meta Analysis- Random Effects Model

Groups		Effect size and 95% interval			Test of null (2-Tail)		Heterogeneity				Tau-squared			
Group	Number Studies	Point estimate	Lower limit	Upper limit	Z-value	P-value	Q-value	df (Q)	P-value	I-squared	Tau Squared	Standard Error	Variance	Tau
**Fixed effect analysis**														
Healthcare/hos	19	1.096	1.073	1.120	8.322	0.000	157.454	18	0.000	88.568	0.046	0.043	0.002	0.215
Payer's	1	4.448	4.376	4.520	180.756	0.000	0.000	0	1.000	0.000	0.000	0.000	0.000	0.000
Total within							157.454	18	0.000					
Total between							10286.863	1	0.000					
**Random effects analysis**														
Healthcare/hos	19	1.481	1.292	1.699	5.627	0.000								
Payer's	1	4.448	4.376	4.520	180.756	0.000								
Total between							244.308	1	0.000					
