# Supplementary material for: The economic burden of antibiotic resistance: A systematic review and meta-analysis
Source: PLoS One. 2023 May 8;18(5):e0285170. doi: 10.1371/journal.pone.0285170 (PMC10166566; doi:10.1371/journal.pone.0285170)
Supplement: S7 Fig — (PDF) [file pone.0285170.s019.pdf]

Supplementary Figure 7. Impact of resistant infections on mortality by healthcare setting

Impact of resistant infections on mortality- by healthcare setting

Study name	Group by Healthcare setting	Odds ratio	Lower limit	Upper limit	p-Value
		Statistics for each study			
Inagaki et al. 2019	Secondary + tertiary hospitals	1.150	1.073	1.233	0.000
Klein et al. 2019	Secondary + tertiary hospitals	1.048	1.022	1.074	0.000
Stewardson et al. 2016	Secondary + tertiary hospitals	1.400	0.929	2.109	0.107
	Secondary + tertiary hospitals	1.105	1.007	1.212	0.036
Thaden et al. 2017	Tertiary hospital	1.280	0.868	1.887	0.212
Judd et al. 2016	Tertiary hospital	2.890	1.149	7.271	0.024
Thatrimontrichai et al. 2019	Tertiary hospital	3.630	2.207	5.971	0.000
Huang et al. 2018	Tertiary hospital	1.322	0.893	1.958	0.163
Maslikowska et al. 2016	Tertiary hospital	3.722	1.154	12.007	0.028
Meng et al. 2017	Tertiary hospital	13.256	1.548	113.508	0.018
Puchter et al. 2018	Tertiary hospital	1.409	0.550	3.609	0.475
Zhen et al. 2020	Tertiary hospital	0.759	0.504	1.143	0.187
Giraldi et al. 2019	Tertiary hospital	4.090	2.055	8.140	0.000
Zhen et al. 2021	Tertiary hospital	1.720	1.256	2.356	0.001
	Tertiary hospital	1.954	1.333	2.863	0.001

Odds ratio and 95% CI

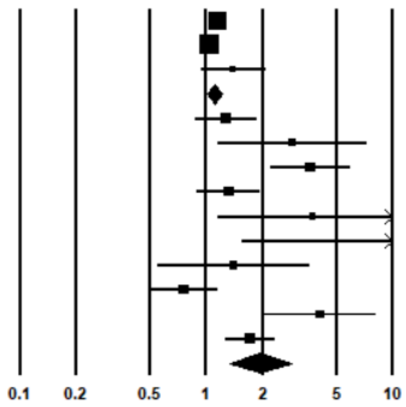

Meta Analysis- Random Effects Model

Groups		Effect size and 95% interval			Test of null (2-Tail)		Heterogeneity				Tau-squared			
Group	Number Studies	Point estimate	Lower limit	Upper limit	Z-value	P-value	Q-value	df (Q)	P-value	I-squared	Tau Squared	Standard Error	Variance	Tau
**Fixed effect analysis**														
Secondary +	3	1.059	1.035	1.084	4.901	0.000	7.911	2	0.019	74.719	0.004	0.007	0.000	0.064
Tertiary hospital	10	1.620	1.379	1.903	5.877	0.000	39.995	9	0.000	77.497	0.251	0.180	0.033	0.501
Total within							47.907	11	0.000					
Total between							26.250	1	0.000					
**Random effects analysis**														
Secondary +	3	1.105	1.007	1.212	2.097	0.036								
Tertiary hospital	10	1.954	1.333	2.863	3.432	0.001								
Total between							8.065	1	0.005					
