# Supplementary material for: The economic burden of antibiotic resistance: A systematic review and meta-analysis
Source: PLoS One. 2023 May 8;18(5):e0285170. doi: 10.1371/journal.pone.0285170 (PMC10166566; doi:10.1371/journal.pone.0285170)
Supplement: S8 Fig — (PDF) [file pone.0285170.s020.pdf]

Supplementary Figure 8. Impact of resistant infections on mortality by income category of country

Impact of resistant infections on mortality by income category of country

Study name	Group by Income category	Odds ratio	Lower limit	Upper limit	p-Value
Thaden et al. 2017	HIE	1.280	0.868	1.887	0.212
Judd et al. 2016	HIE	2.890	1.149	7.271	0.024
Tabak et al. 2020	HIE	1.230	0.410	3.690	0.712
Inagaki et al. 2019	HIE	1.150	1.073	1.233	0.000
Tabak et al. 2019	HIE	1.580	1.137	2.195	0.006
Klein et al. 2019	HIE	1.048	1.022	1.074	0.000
Maslikowska et al. 2016	HIE	3.722	1.154	12.007	0.028
Puchter et al. 2018	HIE	1.409	0.550	3.609	0.475
Stewardson et al. 2016	HIE	1.400	0.929	2.109	0.107
Uematsu et al. 2018	HIE	1.371	1.252	1.501	0.000
Uematsu et al. 2017	HIE	4.448	4.376	4.520	0.000
Giraldi et al. 2019	HIE	4.090	2.055	8.140	0.000
Uematsu et al. 2016	HIE	2.096	1.738	2.528	0.000
Zilberberg et al. 2019	HIE	1.081	0.927	1.259	0.321
	HIE	1.764	1.046	2.974	0.033
Thatrimontrichai et al. 2019	UMIE	3.630	2.207	5.971	0.000
Jiang et al. 2017	UMIE	2.700	1.030	7.075	0.043
Huang et al. 2018	UMIE	1.322	0.893	1.958	0.163
Meng et al. 2017	UMIE	13.256	1.548	113.508	0.018
Zhen et al. 2020	UMIE	0.759	0.504	1.143	0.187
Zhen et al. 2021	UMIE	1.757	1.283	2.406	0.000
	UMIE	1.867	1.107	3.149	0.019

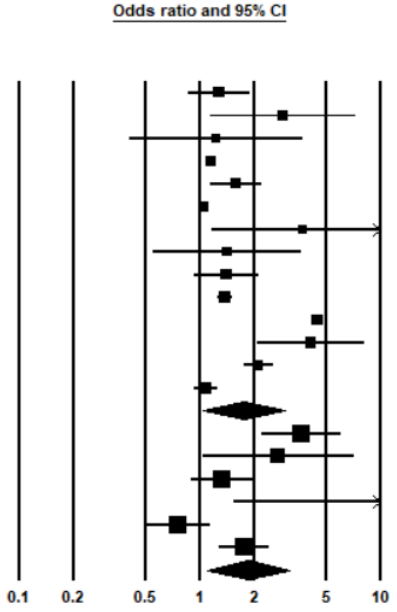

Meta Analysis- Random Effects Model

Groups		Effect size and 95% interval			Test of null (2-Tail)		Heterogeneity				Tau-squared			
Group	Number Studies	Point estimate	Lower limit	Upper limit	Z-value	P-value	Q-value	df (Q)	P-value	I-squared	Tau Squared	Standard Error	Variance	Tau
**Fixed effect analysis**														
HIE	14	2.702	2.667	2.738	149.879	0.000	10384.198	13	0.000	99.875	0.909	1.035	1.071	0.953
UMIE	6	1.577	1.305	1.906	4.718	0.000	29.210	5	0.000	82.883	0.303	0.271	0.073	0.550
Total within							10413.408	18	0.000					
Total between							30.909	1	0.000					
**Random effects analysis**														
HIE	14	1.764	1.046	2.974	2.131	0.033								
UMIE	6	1.867	1.107	3.149	2.343	0.019								
Total between							0.023	1	0.880					
