# Supplementary material for: The economic burden of antibiotic resistance: A systematic review and meta-analysis
Source: PLoS One. 2023 May 8;18(5):e0285170. doi: 10.1371/journal.pone.0285170 (PMC10166566; doi:10.1371/journal.pone.0285170)
Supplement: S9 Fig — (PDF) [file pone.0285170.s021.pdf]

Supplementary Figure 9. Duval and Tweedie's Trim and Fill method for publication bias

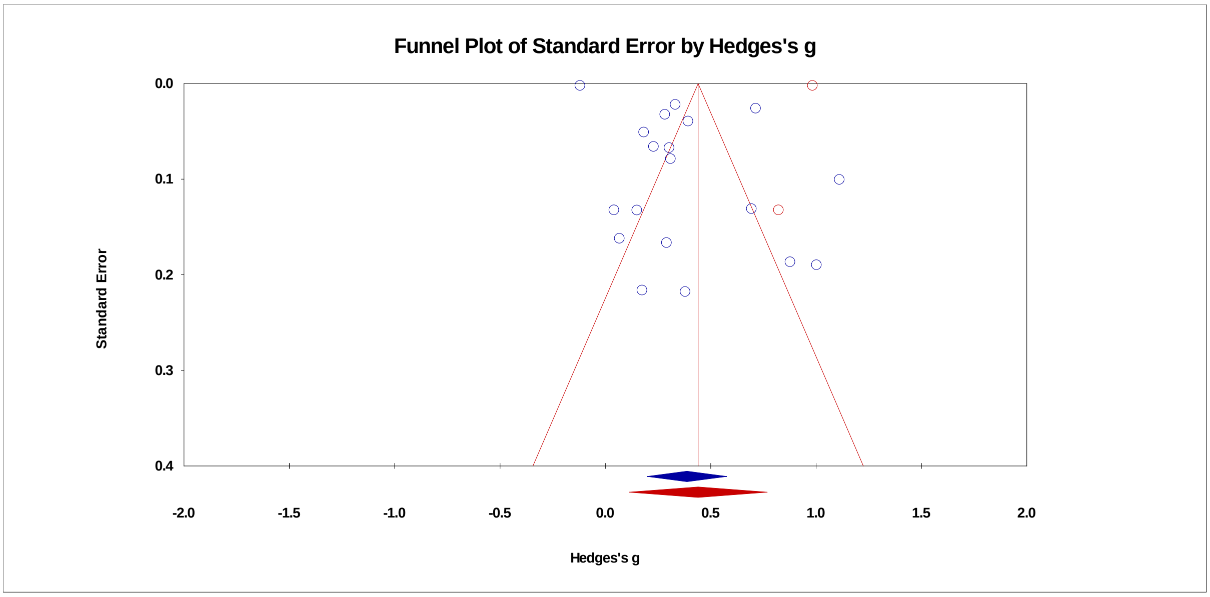

Note: *observed studies are blue in colour and imputed studies are red in colour, Trim and Fill imputed point estimate is 0.440 (95% CI: 0.112- 0.768).*
